# Early Warning Systems and Emergency Declarations During Brazil's Largest Dengue Outbreak

**DOI:** 10.1111/tmi.70153

**Published:** 2026-05-11

**Authors:** Lorena Guadalupe Barberia, Alexandra Crispim Boing, Natália de Paula Moreira, Murilo Motta, Guilherme Pereira Ferreira, Dara Aparecida Vilela Pinto, Luiz Antonio Couto Soares, João de Oliveira Gusmão, Ana Paula Magalhães Brito, Sabrina Almeida, Tatiane C. Moraes de Sousa

**Affiliations:** ^1^ Department of Political Science University of São Paulo, Avenida Professor Luciano Gualberto, Cidade Universitária São Paulo SP Brazil; ^2^ Department of Public Health Federal University of Santa Catarina, Campus Universitário Reitor João David Ferreira Lima, Centro de Ciências da Saúde Floranopolis SC Brazil; ^3^ Institute of International Relations University of São Paulo, Cidade Universitária São Paulo SP Brazil; ^4^ Department of Public Health Emergencies Ministry of Health of Brazil Brasília DF Brazil; ^5^ Department of Social Psychology University of São Paulo, Av. Professor Mello Moraes São Paulo SP Brazil; ^6^ Department of Epidemiology, Institute of Social Medicine State University of Rio de Janeiro Rio de Janeiro RJ Brazil

**Keywords:** Brazil, decentralized health systems, dengue, early warning systems, epidemic risk, public health emergency declarations, public health governance, surveillance

## Abstract

**Objectives:**

Early warning systems (EWSs) are essential for alert, detection, and response to potential public health emergencies. Brazil's 2024 dengue epidemic, the largest ever recorded, offers a critical case for assessing correspondence between timely epidemic risk detection and health governance responsiveness.

**Methods:**

We collected data on dengue cases, deaths, and public health emergency declarations (PHEDs) at federal, state, and municipal levels. We evaluated the timeliness of 2024 emergency declarations by retrospectively mapping observed risk levels across all states and capitals, utilizing the 2023 federal endemic channel guidelines and the updated 2025 risk‐level criteria. Using logistic regressions, we investigated whether epidemic severity was associated with the enactment of a PHED, accounting for primary health coverage and political dynamics that influence local government declarations.

**Results:**

The 2024 epidemic escalated rapidly. Brazil remained at an alert level for 19 consecutive weeks and at an epidemic level for 11, with epidemic risk thresholds reached unusually early. Eleven states and seven capitals issued PHEDs after epidemic thresholds were reached, and an additional five capitals were covered by overlapping state declarations. Despite prolonged epidemic conditions across 18 states and 20 capitals, only 11 states and 7 capitals issued emergency declarations. High‐burden jurisdictions such as Bahia and Belém did not declare emergencies. Logistic regression models show that only the duration at elevated risk levels significantly predicted emergency declarations, while political factors and primary health care coverage did not. That declarations followed prolonged epidemic exposure rather than early detection underscores the need for institutional mechanisms that translate surveillance signals into timely governance responses.

**Conclusions:**

The EWS framework was insufficiently responsive to the rapid dynamics of the 2024 dengue epidemic. Strengthening responsive governance requires adaptive surveillance tools and institutional mechanisms that link early detection to timely, coordinated responses, while accounting for decentralized decision‐making and uneven subnational capacities.

## 1 Background

Dengue is the world's fastest‐growing vector‐borne disease. In Brazil, dengue has become endemic across many regions, with increasing intensity, geographic expansion, and year‐round transmission [1, 2]. On December 1, 2023, the World Health Organization issued its highest alert level (Grade 3) for dengue, and the Americas and the Caribbean experienced record‐breaking outbreaks in 2024 [3, 4]. In 2024, the Pan American Health Organization (PAHO) reported that 90.6% of the 3,405,972 cases reported for the Americas occurred in Brazil [4].

Early warning systems (EWSs) are increasingly critical for outbreak‐prone diseases such as dengue [5, 6, 7]. When effectively operationalized, these systems enable early detection of a dengue outbreak and support anticipating that case increases may exceed expected seasonal patterns, signalling a potential public health emergency. EWSs aim to provide timely alerts that enable the adoption of actions to mitigate a disease's epidemic potential, including enhancing vector control efforts and procuring laboratory surveillance infrastructure and sufficient prophylactics for clinical management of cases. In countries as geographically vast and socioeconomically diverse as Brazil, this requires advanced planning across highly heterogeneous settings, as vector ecology, health system capacity, and administrative resources vary enormously between jurisdictions. If alerts function effectively, measures and resources are mobilized in time to ensure an adequate emergency response—reducing preventable deaths, minimizing harm to population health, and alleviating pressure on the health system.

In countries like Brazil, where multiple vector‐borne diseases are endemic and have experienced successive epidemics of different arboviruses over the past decade, EWSs are increasingly important. However, the effectiveness of EWSs in detecting dengue epidemics is still under evaluation [5, 6, 8, 9]. Although various initiatives [10, 11] have been identified to promote the early detection of arbovirus outbreaks in Brazil, no comprehensive assessment has examined whether evidence of rising risk levels was used to effectively inform administrative decision‐making, particularly the use of emergency declarations as a public health instrument.

Evaluating EWS at the subnational level is especially important for Brazil. Dengue care is primarily provided by primary and secondary health care units, managed by municipalities and states, respectively [12]. Therefore, timely risk detection is crucial for municipalities and states to identify arbovirus outbreaks, as once these epidemics surge, they rapidly strain available resources. The primary and most direct means by which subnational authorities can access additional, more flexible financing is to issue a Public Health Emergency Declaration (PHED), a legal mechanism that provides financial and material resources for local authorities to respond to the emergency. Local governments, in turn, must strategically decide when to issue these announcements, as they are legally required to present evidence that the emergency could endanger public health and overwhelm local infrastructure capacity. In addition, the Ministry of Health's surveillance of dengue outbreaks and activation of the national Emergency Operations Center (EOC) to coordinate the federal response are influenced by alerts issued by subnational authorities.

Early warning and epidemic detection systems should effectively support health system preparedness and response to outbreaks. Such systems must consider not only epidemiological characteristics but also structural and operational constraints of local health systems, including diagnostic and care delivery limitations. Institutional responses should be triggered as early as possible to reduce transmission and prevent healthcare system overload.

In Brazil, the public health system is organized hierarchically, with distinct responsibilities assigned to the municipal, state, and federal levels [13]. The Ministry of Health oversees coordination, technical guidance, and financial and operational support, particularly when subnational capacity is limited [14]. Municipalities are central to healthcare delivery and surveillance, directly implementing response measures, while states coordinate and support municipalities within federal guidelines. These multi‐level governance arrangements create institutional constraints that shape outbreak detection and response. Although detection mechanisms may exist at the federal level, effective risk assessment often depends on local‐level data and contextual knowledge [15].

This study evaluates how the Ministry of Health (MoH)'s 2023 guidelines for endemic‐channel monitoring and the 2025 risk‐level criteria can be applied retrospectively to analyse the 2024 dengue epidemic response. We assessed how epidemiological risk alerts align with administrative decision‐making at the federal, state, and municipal levels. By analysing the temporal concordance between the dengue early‐warning system and PHEDs, we evaluate how the timing and heterogeneity of these actions reflected the interplay between risk‐level detection and governance responsiveness within Brazil's decentralized health system. We also examine whether specific factors reduced local governments' incentives to declare a PHED, while holding epidemiological risk levels constant. Specifically, we assess whether the degree of local population enrolled in the public health system (measured through primary care coverage), political considerations, and overlapping jurisdictions overseeing a local outbreak influenced PHEDs.

## 2 Methods

### 2.1 Data Sources

#### 2.1.1 Dengue Probable Cases and Deaths

Dengue probable cases and deaths were calculated per 100,000 inhabitants from aggregated, anonymized data retrieved from the Notifiable Diseases Information System (SINAN), maintained by the Ministry of Health (MoH) via the DataSus platform [16]. Population data were obtained from the Brazilian Institute of Geography and Statistics (IBGE). Suspect cases exclude cases that were tested and discarded. According to MoH guidelines, probable dengue cases must be registered in the system within 7 days, using both laboratory and clinical‐epidemiological criteria, and dengue deaths must be registered within 24 h. Cases not confirmed within 60 days are excluded and registered as non‐dengue‐related in SINAN.

#### 2.1.2 Contingency Plans for Arboviruses

We reviewed the four national contingency plans for arboviruses issued by the MoH over the past decade (2015, 2022, 2024, and 2025), comparing the risk‐level criteria specified in each (see Supporting Information: Section A.1.2). The 2025 National Contingency Plan for Dengue, Chikungunya, and Zika (NCPDCZ) [17] was adopted for this analysis because it contains the most detailed operational guidance for risk classification and was developed by the MoH during the 2024 epidemic that is the subject of this study.

#### 2.1.3 Public Health Emergency Declarations

PHEDs were collected from the municipal and state governments, with manual retrieval limited to the initial sample already described, and automated searches for health emergency‐related terms using the Google API [18] and Programmable Search Engines applied to all localities [19] The Google search algorithm was tailored to restrict queries to a specified set of domains and automatically extract content from the resulting pages. We also manually searched for PHEDs on the official websites where documents must be published (in Portuguese, *Diário Oficial*) of the municipal, state, and federal governments (see Supporting Information: Section A.1.3). As a robustness check, freedom of information requests were also filed with local governments to verify whether a PHED had been issued.

#### 2.1.4 Primary Health Coverage

Primary health coverage data were obtained from the MoH's e‐Gestor APS platform, using the publicly available “Primary Health Care Coverage” database, with reference to the 2020–2023 National Health Plan (PNS) [20]. This variable refers to the proportion of the population of each state or capital city enrolled in primary care services within the public health system. This indicator is estimated by the MoH and is based on the number of facilities available in the territory, including primary care teams and health units. For the logistic model analyses, binarisation was employed to focus the analysis on meaningful differences across municipalities by assigning a value of 1 to municipalities with coverage at or above the median for state capitals and 0 otherwise.

#### 2.1.5 Local Government Political Alignment and Election Cycles

To assess whether the intention to seek re‐election and ideological alignment between local governments and the federal administration could have influenced emergency declarations, we collected political variables. We employed binarisation to test for a specific threshold effect. For capitals, incumbent mayors seeking re‐election in the 2024 October municipal election were identified using a dummy variable based on data from the Supreme Election Court, except for Brasília, the capital of the federal district, where the governor also serves as mayor and was coded as not seeking re‐election [21] For states, governors were considered aligned with the federal government based on public political positioning at the onset of the Lula III administration [22].

### 2.2 Analytic Approach

#### 2.2.1 Risk Assessment

To estimate weekly risk levels, we first constructed a historical reference distribution—referred to in MoH guidelines as the ‘endemic channel’—against which observed dengue activity in 2024 could be compared. The epidemiological year for arboviruses in Brazil begins in epidemiological week 40/2023 (late September), reflecting the historical seasonality of arbovirus transmission. Based on expert consultation and NCPDCZ guidance, the first 4 weeks of this cycle (epiweeks 40–43) were designated as a calibration period to establish baseline patterns and ensure parameter stability before risk classification begins. From the fifth week onward (epiweek 44), each epidemiological week was hierarchically classified into four risk levels for state capitals, states, and the country by comparing observed dengue incidence and mortality against the endemic channel thresholds in accordance with the 2025 NCPDCZ classification rules until the last week, week 39/2024.

**Level 0 (Normality)** corresponds to baseline conditions, in which observed incidence remains at or below the historical median.

**Level 1 (Mobilization)** is triggered when the weekly incidence of probable dengue cases exceeds the historical median but remains below the upper limit (75th percentile) of the endemic channel for four consecutive weeks. This level signals sustained above‐average activity that has not yet reached historically unusual levels.

**Level 2 (Alert)** is triggered when at least one of the following conditions is met: (1) the weekly case fatality rate among probable cases exceeds 0.05%, signalling that increased severity—even at moderate case counts—warrants heightened vigilance; (2) the weekly incidence surpasses the upper limit of the endemic channel for four consecutive weeks; or (3) the weekly mortality rate increases for four consecutive weeks.

**Level 3 (Epidemic)** is triggered when at least one of the following conditions is met: (1) an exponential increase in incidence—operationalized as a week‐over‐week increase in the natural logarithm of incidence—occurs while incidence remains above the upper limit of the endemic channel; or (2) the mortality rate increases for five consecutive weeks.

#### 2.2.2 Timeliness of PHEDs

The study assessed whether each jurisdiction issued a PHED and, if so, how its timing compared to the first epidemiological week in which Level 3 (Epidemic) risk was reached. Using that week as the temporal benchmark, states and capitals were classified into five categories: (1) preemptive, when the declaration was issued before the week in which Level 3 was first reached; (2) timely, when the declaration was issued during the same week in which Level 3 was first reached; (3) delayed, when the declaration was issued after Level 3 had already been reached; (4) no declaration, when Level 3 was reached but no PHED was issued during the year; and (5) no epidemic, when risk levels did not reach Level 3 during the analysis period.

The first three categories apply exclusively to jurisdictions that reached epidemic levels and classify the governance response along a spectrum from anticipatory action to delayed response. The fourth category identifies jurisdictions where epidemic conditions were met but no administrative action followed. The fifth captures jurisdictions where elevated risk did not escalate to epidemic levels. Considering current Brazilian legislation and contingency plans for arboviruses, this study assesses whether government responses to the 2024 epidemic were timely. States were analyzed for self‐declaration. Since both state and municipal governments can issue emergency declarations that apply to the municipal level, we created a dummy variable equal to 1 when the state declaration preceded the municipal declaration (State Declared Before) and 0 otherwise. For capitals, we considered both self‐declaration and supra‐declaration when a state declaration covers a municipality.

#### 2.2.3 Determinants of PHEDs

Finally, to investigate what distinguished declaring from non‐declaring jurisdictions, we estimated Firth's bias‐reduced logistic regression models for states and capitals separately. Each model controlled for primary health care coverage and political factors, and used one of three alternative measures of epidemic severity as the primary explanatory variable: (1) the maximum number of consecutive weeks at Alert level; (2) the maximum number of consecutive weeks at Epidemic level; or (3) the maximum number of consecutive weeks at either Alert or Epidemic level. The purpose of these models is to test if sustained exposure to elevated risk—rather than threshold exceedance alone—differentiates jurisdictions that declared emergencies from those that did not. Models were estimated in R (v. 4.5.2) using the ‘logistf’ package (v. 1.26.1), with confidence intervals as recommended by Heinz et al. [23, 24].

## 3 Results

### 3.1 Risk Assessment: Dengue Epidemic Risk Levels and Their Duration

**FIGURE 1:**
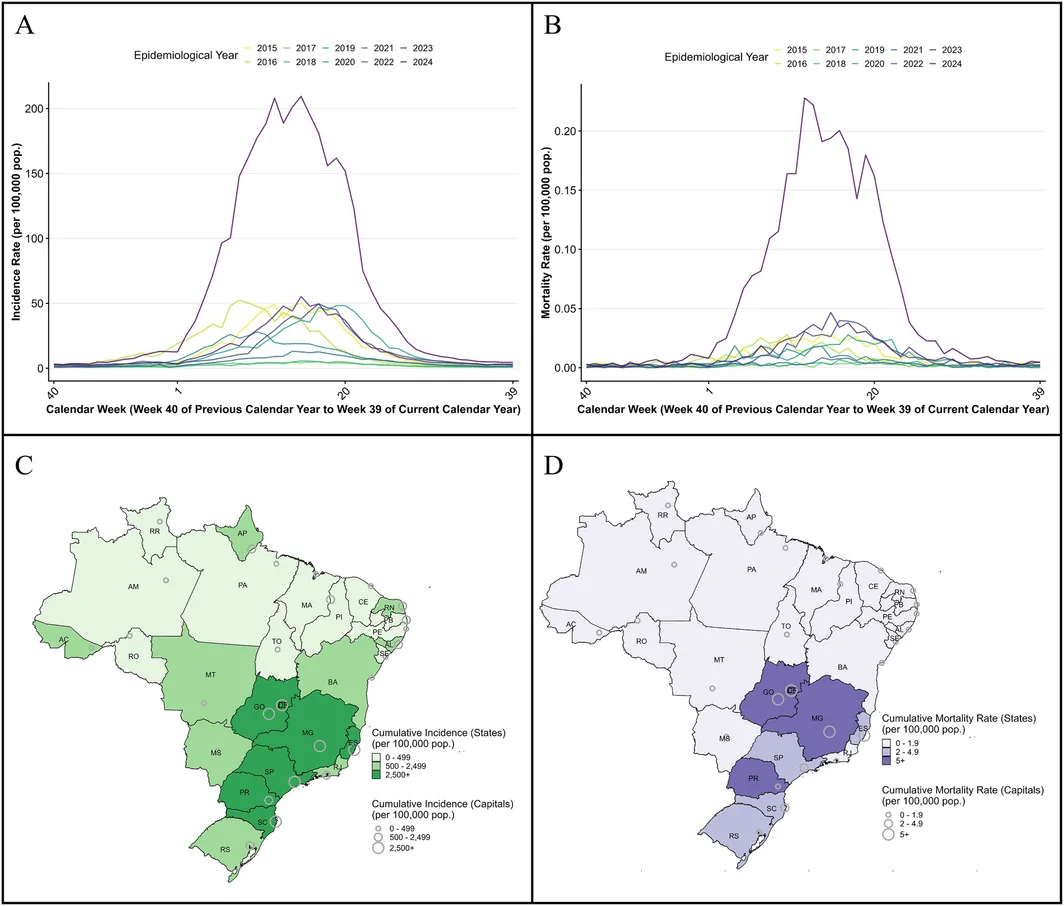
Incidence rate (Panel A) and mortality rate (Panel B) of dengue cases in Brazil, epidemiological years 2015–2024 (2014w40 to 2024w39), and cumulative incidence (Panel C) and cumulative mortality rate (Panel D) across Brazilian states and capitals in epidemiological year 2024 (2023w40 to 2024w39). AC = Acre; AL = Alagoas; AP = Amapá; AM = Amazonas; BA = Bahia; CE = Ceará; DF = Distrito Federal; ES = Espírito Santo; GO = Goiás; MA = Maranhão; MT = Mato Grosso; MS = Mato Grosso do Sul; MG = Minas Gerais; PA = Pará; PB = Paraíba; PR = Paraná; PE = Pernambuco; PI = Piauí; RJ = Rio de Janeiro; RN = Rio Grande do Norte; RS = Rio Grande do Sul; RO = Rondônia; RR = Roraima; SC = Santa Catarina; SP = São Paulo; SE = Sergipe; TO = Tocantins.

Between epidemiological weeks 2023w40 and 2024w39, Brazil recorded an incidence rate of 3289 dengue cases per 100,000 inhabitants and a mortality rate of 3.13 deaths per 100,000 inhabitants (Figure 1—Panels A and B). Considering the 2024 outbreak, the cumulative incidence was highest in the Federal District, Goias, Espirito Santo, Minas Gerais, Sao Paulo, Parana and Santa Catarina (FIgure 1—Panel C). Of these states, the Federal District, Goias, Minas Gerais, and Parana experienced the highest mortality rates (Figure 1—Panel D).

**FIGURE 2:**
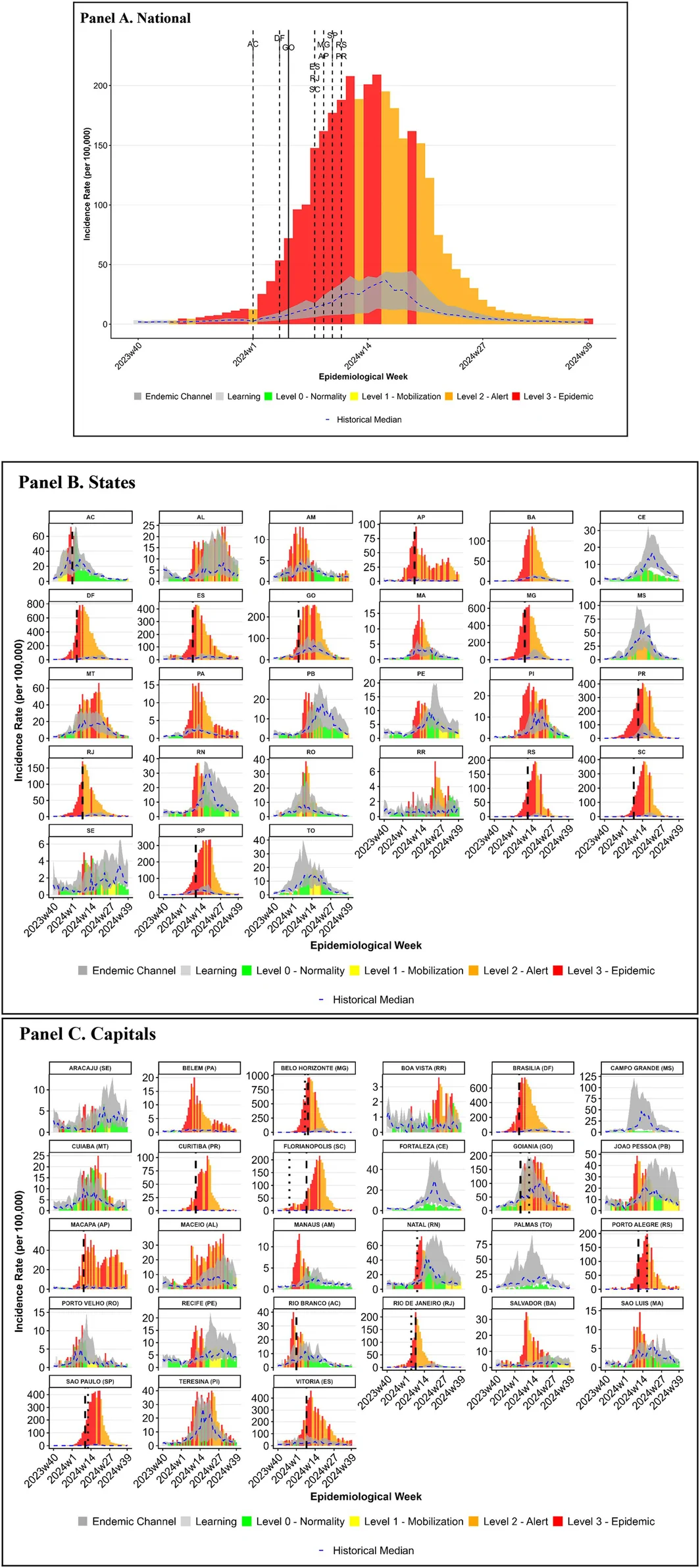
Dengue outbreak risk levels in 2024 by epidemiological week in Brazil (Panel A), States (Panel B), and Capitals (Panel C). The dengue control diagram presents median lines, upper and lower limits, and risk levels are presented in green (normal), yellow (mobilization), orange (alert), and red (epidemic). (A) The black line represents the timing of the establishment of the EOC by the MOH. The dashed lines are state public health emergency declarations. (B) The dashed lines are the state public health emergency declaration. The white area in the case of Ceará is because the incidence is below the endemic channel. (C) Dotted lines are Municipal Government public health emergency declarations. Dashed Lines are State Government public health emergency declarations that also apply to the State Capital. The white area in the case of Campo Grandes, Fortaleza and Palmas is because the incidence is below the endemic channel.

All Brazilian states (Figure 2, Panel B) reached the Alert level. Except for Ceará, Mato Grosso do Sul, and Tocantins, all Brazilian states reached Epidemic levels. Similarly, all state capitals (Figure 2—Panel C) reached the Alert level. Only Campo Grande (MS), Fortaleza (CE), and Palmas (TO) did not evolve to Epidemic levels. The severity of the epidemic can also be assessed by the number of weeks under higher risk levels (Alert or Epidemic). Eighteen states (67%) experienced Alert or Epidemic levels for 10 or more consecutive weeks. The duration of high‐risk conditions varied significantly across the country. Six states (MG, PR, RJ, RS, SC, and SP) maintained high‐risk status for 48 weeks. Conversely, states such as Alagoas and Pernambuco experienced the shortest durations, totaling only 10 weeks. The remaining states showed moderate persistence, with a median duration of 32 weeks.

Twenty (74%) state capitals experienced Alert or Epidemic levels for 10 or more consecutive weeks. Of these, 20% (*n* = 4) remained at Alert or Epidemic levels for the entire 48‐week period, while 35% (*n* = 7) faced significant durations exceeding 30 weeks. The remaining 45% (*n* = 9) experienced higher risk levels for durations ranging from 10 to 28 weeks. The shortest durations within this high‐risk group were observed in Manaus and João Pessoa (10 weeks).

### 3.2 Public Health Emergency Declaration Responsiveness

Figure 2, Panel A, depicts vertical dashed lines indicating when specific states declared emergencies, and a black vertical line for the epiweek (2024w5) when the MoH established an EOC. Across states and capitals, PHEDs were issued only after the Epidemic Level threshold had been reached, classifying them as delayed responses. No instances of timely declarations were identified (see Table 1).

**TABLE 1:** Final classification of state capitals and states for 2024 epidemic.

Cases	Capitals	States
*Preemptive* (Epiweek Level 3_t_ > Epiweek Emergency Declaration_t_)	None	None
*Timely* (Epiweek Level 3_t_ = Epiweek Emergency Declaration_t_)	None	None
*Delayed* (Epiweek Level 3_t_ < Epiweek Emergency Declaration_t_)	Belo Horizonte (MG)	Acre
	Brasília (DF)^a^	Amapá
	Curitiba (PR)^a^	Distrito Federal
	Florianópolis (SC)	Espírito Santo
	Goiânia (GO)	Goiás
	Macapá (AP)^a^	Minas Gerais
	Natal (RN)	Paraná
	Porto Alegre (RS)	Rio de Janeiro
	Rio Branco (AC)^a^	Rio Grande do Sul
	Rio de Janeiro (RJ)	Santa Catarina
	São Paulo (SP)	São Paulo
	Vitória (ES)^a^	
*No declaration* (Despite reaching Level 3, no emergency declaration was issued)	Aracaju (SE)	Alagoas
	Belém (PA)	Amazonas
	Boa Vista (RR)	Bahia
	Cuiabá (MT)	Maranhão
	João Pessoa (PB)	Mato Grosso
	Maceió (AL)	Pará
	Manaus (AM)	Paraíba
	Porto Velho (RO)	Pernambuco
	Recife (PE)	Piauí
	Salvador (BA)	Rio Grande do Norte
	São Luís (MA)	Rondônia
	Teresina (PI)	Roraima
		Sergipe
*No epidemic* (Risk levels did not reach Level 3)	Campo Grande (MS)	Ceará
	Fortaleza (CE)	Mato Grosso do Sul
	Palmas (TO)	Tocantins

^a^ Capital did not issue a self‐declaration; classified as delayed based on state‐level (supra) declaration.

Eleven states issued PHEDs in 2024 epidemic: Acre, Amapá, Federal District, Espírito Santo, Goiás, Minas Gerais, Paraná, Rio de Janeiro, Rio Grande do Sul, Santa Catarina, and São Paulo, representing all Brazilian regions. These declarations occurred in two clusters: 2024w1 to 2024w5 (Acre, Federal District, Goias) and 2024w8 to 2024w11 (Amapá, Espírito Santo, Minas Gerais, Paraná, Rio de Janeiro, Rio Grande do Sul, Santa Catarina, and São Paulo). In both clusters, public health emergencies were issued after epidemic conditions had already been reached. Considering the week in which a state reached level 2 risk or higher, declarations were made from 3 to 19 weeks afterwards.

Seven capitals issued PHEDs: Belo Horizonte, Florianópolis, Goiânia, Natal, Porto Alegre, Rio de Janeiro, and São Paulo. In six of these capitals, states also issued declarations. However, the state of Rio Grande do Norte, whose capital is Natal, did not issue a PHED. Considering supra declarations since five state capitals were covered by state‐level declarations: Brasília (Federal District), Curitiba (Paraná), Macapá (Amapá), Rio Branco (Acre), and Vitória (Espírito Santo), the total number of capitals with PHEDs is 12. One capital, Florianópolis, issued two PHEDs (2023w48 and 2024w8). In the analysis, the date of the first of these declarations was used, and the second was excluded. Florianópolis was also the first to issue a PHED (2023w48), while Porto Alegre was the last of the capitals to issue a declaration (2024w17). Declarations were made from 4 to 25 weeks after risk level 2 or higher was reached.

The first week of the Alert risk level was relatively earlier for the states of the Central‐West, Southeast, and South. There is greater variation among the states of the North and Northeast regions regarding the period in which the first week of Alert occurs (see Supporting Information: Section A.2.3.4). Delayed declarations were mostly concentrated in the South and Southeast of Brazil, while North and Northeast states did not issue PHEDs (see Supporting Information: Section A.2.3.5). Additionally, states and capitals that issued PHEDs experienced a greater number of consecutive weeks classified as Alert or Epidemic (see Supporting Information: Sections A.2.3.6 and A.2.3.7).

### 3.3 Determinants of PHEDs

Firth's bias‐reduced logistic regressions indicate that sustained exposure to elevated risk—rather than threshold exceedance alone—differentiated jurisdictions that declared emergencies from those that did not (see Table 2). For states, each additional consecutive week at Alert level was associated with a 23% increase in the odds of issuing a PHED (OR = 1.23; 95% CI 1.05, 1.56); substituting weeks at Epidemic level yielded a stronger association (OR = 1.35; 95% CI 1.10, 2.16); and using the combined measure of weeks at either level produced consistent results (OR = 1.12; 95% CI 1.05, 1.27). The pattern for capitals was analogous across all three specifications: Alert (OR = 1.47; 95% CI 1.11, 2.25), Epidemic (OR = 1.35; 95% CI 1.06, 2.06), and combined (OR = 1.13; 95% CI 1.05, 1.27).

**TABLE 2:** Firth's bias‐reduced logistic regression: Dependent variable—Emergency declaration.

Characteristic	Model 1			Model 2			Model 3		
	OR	95% CI	*p*	OR	95% CI	*p*	OR	95% CI	*p*
Panel A: States									
Max consecutive weeks level 2 (alert)	1.23	1.05, 1.56	0.009						
Max consecutive weeks level 3 (epidemic)				1.35	1.10, 2.16	< 0.001			
Max consecutive weeks level 2 or 3							1.12	1.05, 1.27	< 0.001
Primary care coverage (above median)	0.53	0.08, 3.38	0.50	0.42	0.04, 3.83	0.43	0.52	0.04, 5.37	0.58
President–Governor alignment	0.35	0.04, 2.30	0.27	0.25	0.01, 1.93	0.19	0.17	0.01, 1.55	0.12

Characteristic	Model 4			Model 5			Model 6		
	OR	95% CI	*p*	OR	95% CI	*p*	OR	95% CI	*p*
Panel B: Capitals									
Max consecutive weeks level 2 (alert)	1.47	1.11, 2.25	0.002						
Max consecutive weeks level 3 (epidemic)				1.35	1.06, 2.06	0.010			
Max consecutive weeks level 2 or 3							1.13	1.05, 1.27	< 0.001
Primary care coverage (above median)	0.30	0.03, 1.92	0.21	0.87	0.16, 5.22	0.87	0.75	0.08, 6.92	0.78
Mayor seeking reelection	0.59	0.05, 5.71	0.64	0.70	0.11, 4.56	0.71	0.17	0.00, 2.38	0.20

*Note: N* = 27 for both panels. Panel A controls for primary care coverage and president–governor alignment. Panel B controls for primary care coverage and whether the mayor was seeking reelection.

Abbreviations: CI = Confidence Interval; OR = Odds Ratio.

## 4 Discussion

The exceptional magnitude of the 2024 epidemic in Brazil, in terms of both cases and deaths, was preceded by an unprecedented sequence of epidemic years and accompanied by a shift in hotspot areas, with higher incidence observed in the country's south‐central regions [25]. These factors point to a broader shift in the dynamics of arboviral transmission in Brazil, driven in part by climate‐related phenomena, such as rising temperatures and humidity levels and intensified El Niño effects [26, 27], as well as by the persistence of conditions favourable to the proliferation of the main vector of dengue and other arboviruses in Brazil, *Aedes aegypti* [28] Compounding this scenario is the circulation of all four dengue serotypes (DENV‐1, −2, −3, and −4) and substantial socioenvironmental diversity, which increases population susceptibility to both infection and severe clinical presentations [29].

The 2024 dengue epidemic underscores the need to strengthen Early Warning Systems (EWS) in Brazil to ensure the timely identification of epidemics and other public health emergencies, thereby mitigating their impacts, including disease spread and increases in deaths and cases. In scenarios where a potential epidemic is not detected early and the health system shows signs of saturation, it is urgent to have the flexibility to allocate resources appropriately. In the Brazilian context, the primary legal instrument that enables such flexibility is the Public Health Emergency Declaration (PHED). Our findings indicate that neither the severity of the epidemic nor the unprecedented number of cases and deaths is sufficient to prompt the use of this instrument by policymakers at different levels of government (state and municipalities). This suggests that technical criteria alone are insufficient to trigger the declaration of public health emergencies.

Our results also demonstrate that neither the use of control charts nor the widespread evidence that Brazilian states and capitals had entered alert and/or epidemic stages was sufficient to trigger the PHED. This points to structural and institutional factors shaping decision‐making beyond epidemiological evidence alone. One plausible explanation is that managers assumed that existing resources would suffice or that support from higher levels of government (state or federal) would be mobilized if necessary. This assumption reflects the institutional design of the Brazilian public health system, in which the MoH and, subsequently, state governments are formally responsible for coordinating emergency responses. However, this arrangement has previously proven limited, as evidenced during the COVID‐19 pandemic, when federal inaction effectively transferred responsibility to states and municipalities without providing the necessary organizational, financial, or decision‐making capacity to ensure an effective response [30].

Local outbreaks may overwhelm specific municipalities, but state governments may not issue a PHED, as their statewide network may still be able to handle increased demand. Similarly, municipal governments may hesitate to declare emergencies because overall state conditions may not yet justify an escalation. In addition, state capitals generally have greater health services capacity and managerial autonomy than other municipalities within the same state, partly due to the concentration of health workers and resources within their territories. This disparity is reflected in the distribution of physicians, for example, with capitals having a much higher physician‐to‐population ratio (6.97 per 1000 inhabitants) than non‐metropolitan municipalities (1.9 per 1000) [31]. However, this greater capacity did not translate into earlier or more frequent PHED declarations.

Furthermore, contexts of heightened political polarization and electoral competition may hinder the translation of early warning signals into PHEDs. In the absence of political alignment, opposing actors may be more inclined to assign blame to one another, while frequent turnover among local health public managers can further weaken both technical and political responses to public health emergencies [32]. During the 2024 dengue epidemic, approximately half of Brazil's governors were politically opposed to the federal administration, while many capital‐city mayors were simultaneously running for re‐election. Under such conditions, local authorities may have been more reluctant to issue PHEDs, as doing so could signal an inability to manage the crisis and imply that higher levels of government (state or federal) were better equipped to respond. This dynamic may create political incentives to delay or avoid emergency declarations, particularly when such responses could be leveraged for electoral advantage. Although political alignment—whether between governors and the federal government or linked to municipal re‐election efforts—was not statistically significant in our analysis, this finding likely reflects limited statistical power due to the small sample size (27 states and their capitals), rather than the absence of a meaningful relationship between political dynamics and public health emergency responses.

Several directions for future research emerge. No corrections for deduplication, reclassification, or reporting delays were applied to the weekly counts of probable cases and deaths, as the study aimed to reflect the real‐time information environment under which decision‐makers operated, including the inherent uncertainty embedded in notification systems.

The NCPDCZ framework was developed for real‐time epidemic monitoring and accordingly relies on nowcasting to adjust for reporting delays and incomplete clinical or laboratory confirmation. In the present study, however, cases had already been investigated and classified at the time of analysis, rendering nowcasting unnecessary. The original epidemiological indicators and classification logic were therefore preserved, with only operational adjustments required to adapt the framework for retrospective application. A detailed description of this adaptation is provided in the Supporting Information (Section A.2.1). Future research could apply nowcasting techniques and retrospectively cleaned data to assess whether accounting for these lags yields distinct findings regarding the timing and likelihood of PHED declarations.

The endemic channel was constructed using data from 2014 to 2023, a period in which many Brazilian states experienced dengue epidemics in multiple years (2014, 2015, 2016, 2019, 2022, and 2023). Although including epidemic years in the baseline may elevate historical thresholds, excluding them would reduce the dataset by more than 5 years, undermining the statistical basis for constructing the endemic channel. Moreover, this exclusion is not mandated by the MoH's Technical Note. Future studies should explore alternative approaches, such as down‐weighting or smoothing epidemic years, to assess the sensitivity of risk‐level classifications to baseline construction.

This study focused on states and their capitals. Future studies should collect data for a larger cross‐sectional and cross‐temporal dataset to investigate whether an emergency declaration was more (or less) likely to be issued in smaller and medium‐sized cities. There is evidence that cooperation between governments may explain why some governments did not declare. Indeed, of the seven capitals that issued PHEDs, only three did so after states had declared an emergency. Efforts should also be directed at examining how political misalignments between health secretaries and incumbent leaders [32] contribute to delays in issuing PHEDs and identifying how horizontal and vertical accountability jointly and separately drive PHED adoption.

## Funding

This work was supported as part of the Advanced Warning and Response Exemplars (AWARE) Project, coordinated by the Pandemic Center at Brown University, which was funded by Gates Ventures (008975) for Brazil.

## Conflicts of Interest

The authors declare no conflicts of interest.

## Supporting information


**Data S1:** Appendix.

## Data Availability

The data that support the findings of this study are openly available in the Harvard Dataverse of the corresponding author.
