# Supplementary material for: Early Warning Systems and Emergency Declarations During Brazil's Largest Dengue Outbreak
Source: Trop Med Int Health. 2026 May 11;31(9):1183–93. doi: 10.1111/tmi.70153 (PMC13533633; doi:10.1111/tmi.70153)
Supplement: Supplementary file 1 [file TMI-31-1183-s001.pdf]

Supplementary Materials:

A Retrospective Assessment of Early Warning Systems and Emergency Declarations during Brazil's Largest Dengue Outbreak

March 30, 2026

Contents

A.1 Data Sources and Visualizations 1
A.1.1 Dengue probable cases and deaths . . . 1
A.1.1.1 Incidence of Dengue in Brazil, Epidemiological Years 2015-2024 . . . 2
A.1.1.2 Incidence of Dengue in 26 States and the Federal District, Epidemiological Years 2015-2024 . 3
A.1.1.3 Incidence of Dengue in 26 State Capitals and Brasília, Epidemiological Years 2015-2024 . . . 4
A.1.1.4 Incidence of Dengue in 26 States and the Federal District, 2023w40 to 2024w39 . . . 5
A.1.1.5 Incidence of Dengue in 26 State Capitals and Brasília, 2023w40 to 2024w39 . . . 6
A.1.1.6 Map of Cumulative Dengue Incidence, per States and Capitals, 2023w40-2024w39 . . . 7
A.1.1.7 Deaths Due to Dengue in Brazil, Epidemiological Years 2015-2024 . . . 8
A.1.1.8 Deaths per Year Due to Dengue in 26 States and the Federal District, Epidemiological Years 2015-2024 . . . 9
A.1.1.9 Deaths per Year Due to Dengue in 26 State Capitals and Brasília, Epidemiological Years 2015-2024 . . . 10
A.1.1.10 Deaths Due to Dengue in 26 States and the Federal District, 2023w40 to 2024w39 . . . 11
A.1.1.11 Deaths Due to Dengue in 26 State Capitals and Brasília, 2023w40 to 2024w39 . . . 12
A.1.1.12 Map of Cumulative Dengue Death Rate, per States and Capitals, 2023w40-2024w39 . . . 13
A.1.2 Brazilian Arboviruses Contingency Plans, 2014-2025 . . . 14
A.1.3 Public Health Emergency Declarations . . . 24
A.1.3.1 Sources and List of terms used to search for health emergency declarations . . . 24
A.1.3.2 Country Level . . . 26
A.1.3.3 State Level . . . 26
A.1.3.4 State Capitals . . . 27
A.1.4 Primary Health Coverage . . . 28
A.1.5 Local Government Political Alignment and Election Cycles . . . 29
A.1.5.1 Governors Aligned with Lula III Administration . . . 29
A.1.5.2 Re-election of Mayors in State Capitals . . . 30

A.2 Analytical Approach 31
A.2.1 Protocol for Risk Assessment of Dengue Epidemic Potential . . . 31
A.2.1.1 Endemic Channel . . . 32
A.2.1.2 Risk Level Classification . . . 33
A.2.2 Summary Table of Risk Assessment in Epidemiological Year 2024 (2023w40 - 2024w39) . . . 35
A.2.2.1 Country Level . . . 35
A.2.2.2 State Level . . . 35
A.2.2.3 State Capitals Level . . . 36
A.2.3 Visualizing Risk Assessment and Emergency Declarations in 2024 . . . 38

A.2.3.1 Country-wide Risk Assessment of Dengue Outbreak and Emergency Declarations in 2023w40-2024w39 . . . 38
A.2.3.2 State-level Risk Assessment of Dengue Outbreak and States Emergency Declarations, 2023w40-2024w39 . . . 39
A.2.3.3 State Capital-level Risk Assessment of Dengue Outbreak and Emergency Declarations, 2023w40-2024w39 . . . 40
A.2.3.4 Map of First Epidemic Week, 2023w40-2024w39, States and Capitals . . . 41
A.2.3.5 Map of Lag between First Epidemic Week and Emergency declaration, 2023w40-2024w39, States and Capitals . . . 42
A.2.3.6 Correlation Between Max. Consecutive Weeks under 'Epidemic' and 'Alert' Risk Level and Emergency Declaration, 2023w40-2024w39, States . . . 43
A.2.3.7 Correlation Between Max. Consecutive Weeks under 'Epidemic' and 'Alert' Risk Level and Emergency Declaration, 2023w40-2024w39, Capitals . . . 44
A.2.4 Classification of Emergency Declaration Timeliness of State and States Capitals for 2024 Epidemic . 45
A.2.5 Epidemic Severity and Public Health Declarations . . . 46
A.2.5.1 Empirical Strategy . . . 46
A.2.5.2 Regression Results . . . 46

List of Figures

1 Incidence of Dengue in Brazil, 2015–2024 2
2 Incidence of Dengue per State, 2015-2024 3
3 Incidence of Dengue in State Capitals, 2015-2024 4
4 Incidence of Dengue per State, 2023w40 to 2024w39 5
5 Incidence of Dengue in State Capitals, 2023w40 to 2024w39 6
6 Map of Cumulative Dengue Incidence, per States and Capitals, 2023w40-2024w39 7
7 Deaths Due to Dengue in Brazil, 2015-2024 8
8 Deaths per Year Due to Dengue, per State, 2015-2024 9
9 Deaths per Year Due to Dengue in State Capitals, 2015-2024 10
10 Incidence of Dengue per State, 2023w40 to 2024w39 11
11 Incidence of Dengue in State Capitals, 2023w40 to 2024w39 12
12 Map of Cumulative Dengue Death Rate, per States and Capitals, 2023w40 to 2024w39 13
13 Country-wide Risk Assessment of Dengue Outbreak and Emergency Declarations, 2023w40 to 2024w39 38
14 Risk Assessment of Dengue Outbreak and States Emergency Declarations, 2023w40 to 2024w39 . . . 39
15 Municipality-level Risk Assessment of Dengue Outbreak and Emergency Declarations, 2023w40 to 2024w39 40
16 Map of First Epidemic Week, 2023w40-2024w39, States and Capitals 41
17 Map of Lag between First Epidemic Week and Emergency declaration, 2023w40-2024w39, States and Capitals 42
18 Correlation Between Max. Consecutive Weeks under 'Epidemic' and 'Alert' Risk Level and Emergency Declaration, 2023w40-2024w39, States 43
19 Correlation Between Max. Consecutive Weeks under 'Epidemic' and 'Alert' Risk Level and Emergency Declaration, 2023w40-2024w39, Capitals 44

List of Tables

1 List of contingency plans and related documents by disease for federal government. 14
2 List of contingency plans and related documents by disease for state governments. 14
3 List of contingency plans and related documents by disease for municipal governments. 18
4 Risk level classification and endemic channel adoption in the most recent contingency plans from state and municipal governments 22
5 State-wide Dengue-Related Emergency Declarations (chronological order) 26
6 Municipal-specific Emergency Declarations for Dengue (chronological order) 27
7 Governor–President Alignment (Brazil, 2023) 29
8 Incumbent Mayors Seeking Reelection (Brazil Capitals, 2024 Election) 31
9 List of First Week and Max. consecutive weeks for level 2 and 3 - Country Level 35
10 List of First Week and Max. consecutive weeks for level 2 and 3 - State Level 35
11 List of First Week and Max. consecutive weeks for level 2 and 3 - State Capital Level 36
12 Final Classification of State Capitals and States for 2024 Epidemic 45
13 Firth's Bias-Reduced Logistic Regression: Dependent Variable – Emergency Declaration 47

A.1 Data Sources and Visualizations

A.1.1 Dengue probable cases and deaths

Dengue probable cases and deaths were calculated per 100,000 inhabitants using aggregated anonymized data retrieved from the Notifiable Diseases Information System (SINAN), maintained by the Ministry of Health (MoH)12. Population data were obtained from the Brazilian Institute of Geography and Statistics (IBGE). According to MoH guidelines, probable dengue cases must be registered in the system within seven days, considering both laboratorial and clinical-epidemiological criteria, and dengue deaths within 24 hours.13 However, confirmed cases and deaths are notified with a delay, depending on the length of time required for case closure. Cases that are not confirmed within 60 days are excluded and registered as not dengue-related in the SINAN. The data were retrieved from https://datasus.saude.gov.br/acesso-a-informacao/doencas-e-agravos-de-notificacao-de-2007-em-diante-sinan/.

Dengue cases and death data were collected from the Datasus website (Notifiable Diseases Information System-SINAN) as CSV files from 2014 to 2024. Separate csv files were downloaded for each year for: (i) suspect cases at the municipal level and organized by the notification epidemiological week, (ii) deaths with the evolution field marked with the cause of death if it was due to the specific disease (dengue). Suspect cases exclude cases that were tested and discarded. The complete dataset encompasses dengue incidence and deaths data for Brazil from the first epidemiological week of 2014 through the 52nd epidemiological week of 2024, as per DataSUS definition of epidemiological week. The risk analysis analysis adopts a specialized arbovirus epidemiological year, defined as the period from week 40 of the previous calendar year (the first week of winter in the Southern Hemisphere) to week 39 of the current calendar year. Consequently, the data utilized begins in the 40th week of 2014, which marks the start of the 2015 arbovirus epidemiological year.

For 2020, the database in the Datasus website was not marked in epidemiological weeks, so the data was collected directly via ftp from the ”SINAN ABERTO” and tabulated. For the state of Espírito Santo, suspect cases from 2021 to 2024 were missing in the Datasus database. Therefore, the data from 2021 to 2022 were retrieved from the state’s ”Painel de Vigilância” website. Cases from 2023 to 2024 were sent by e-mail by the Health Vigilance Sub-secretary. Death cases were obtained by the entry for case evolution.

A.1.1.1 Incidence of Dengue in Brazil, Epidemiological Years 2015-2024

Figure 1: Incidence of Dengue in Brazil, 2015–2024

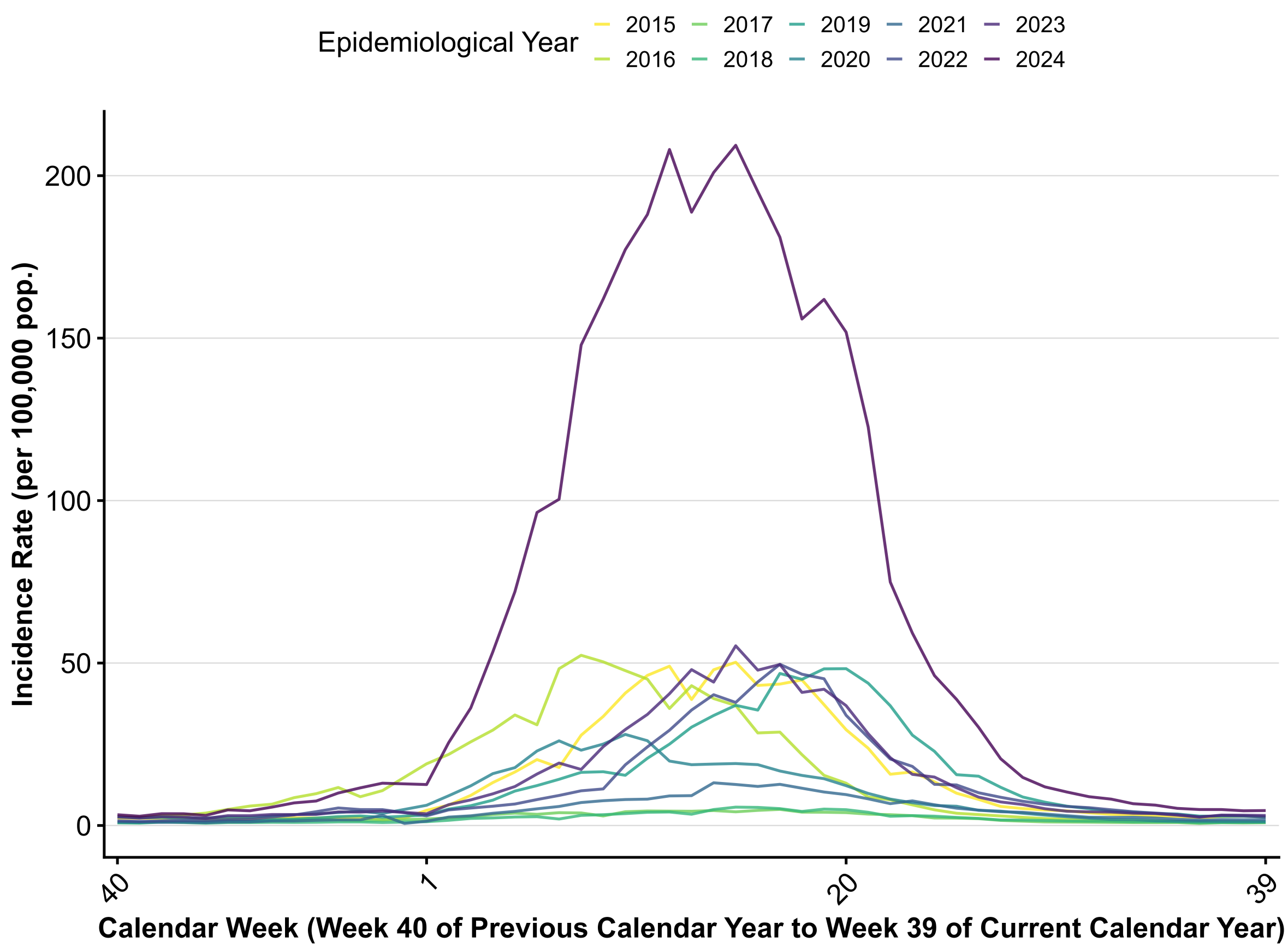

Source: Authors' elaboration based on data from the Notifiable Diseases Information System (SINAN)

A.1.1.2 Incidence of Dengue in 26 States and the Federal District, Epidemiological Years 2015-2024

Figure 2: Incidence of Dengue per State, 2015-2024

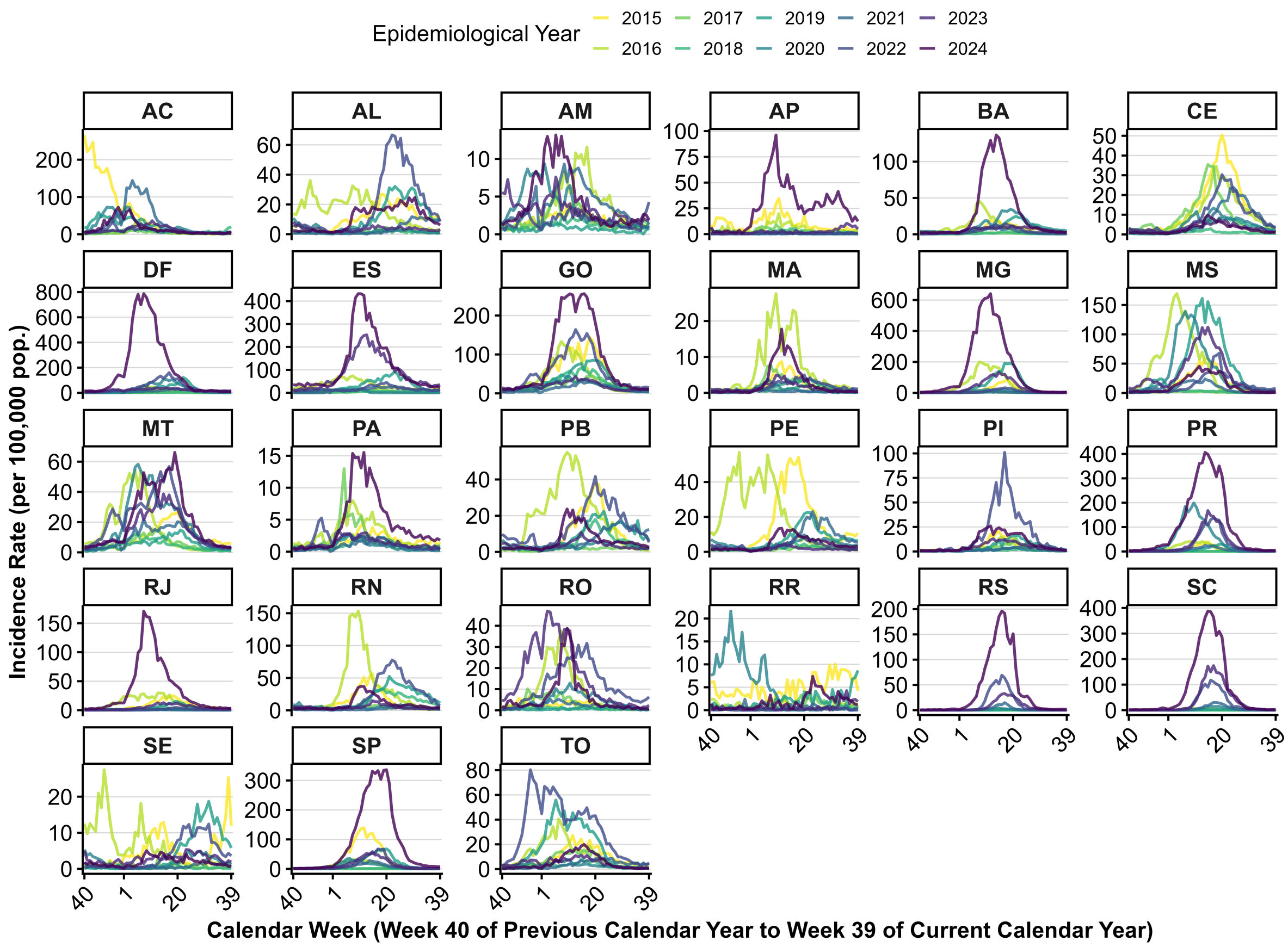

Source: Authors' elaboration based on data from the Notifiable Diseases Information System (SINAN)

A.1.1.3 Incidence of Dengue in 26 State Capitals and Brasília, Epidemiological Years 2015-2024

Figure 3: Incidence of Dengue in State Capitals, 2015-2024

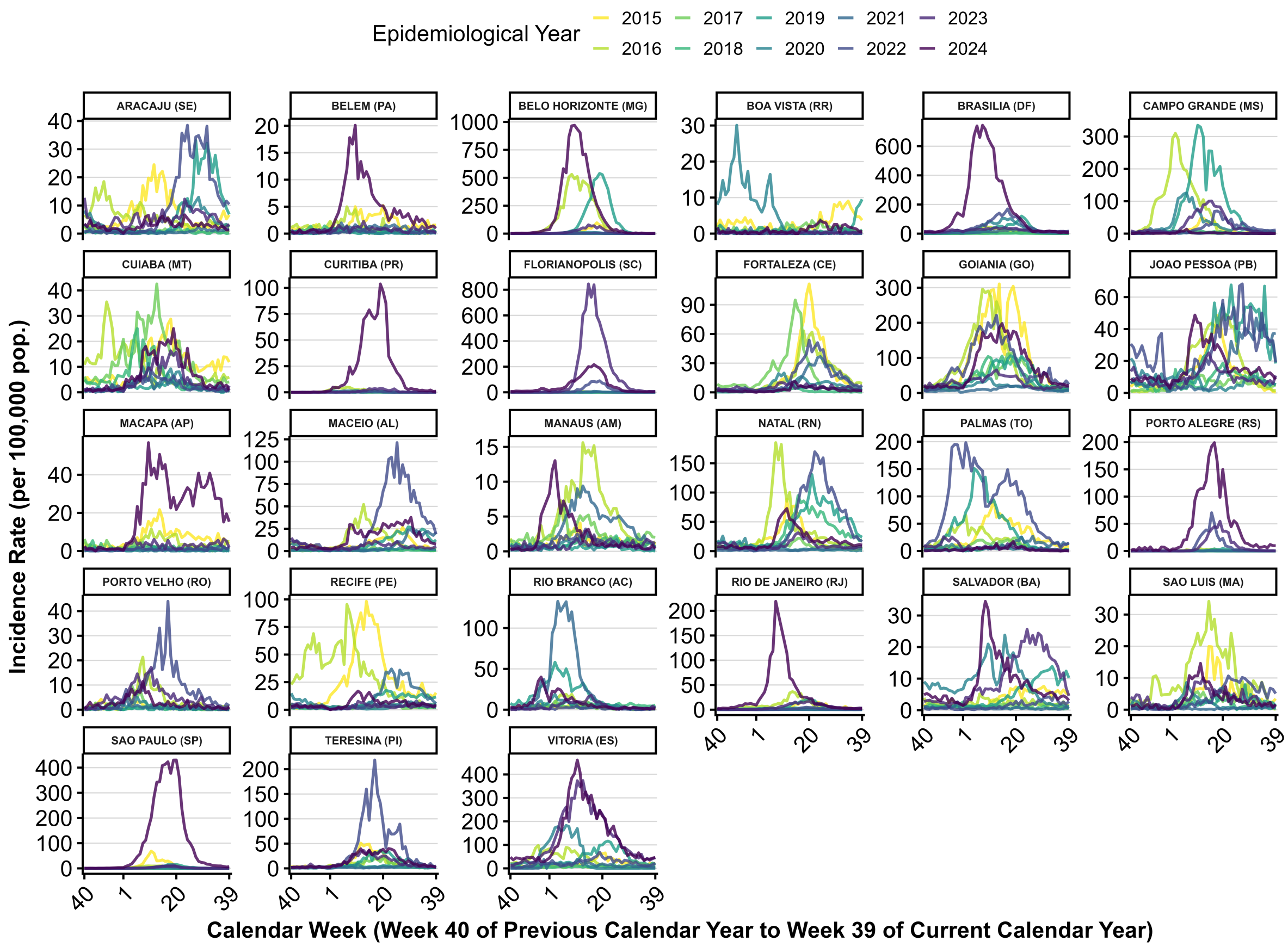

Source: Authors' elaboration based on data from the Notifiable Diseases Information System (SINAN)

A.1.1.4 Incidence of Dengue in 26 States and the Federal District, 2023w40 to 2024w39

Figure 4: Incidence of Dengue per State, 2023w40 to 2024w39

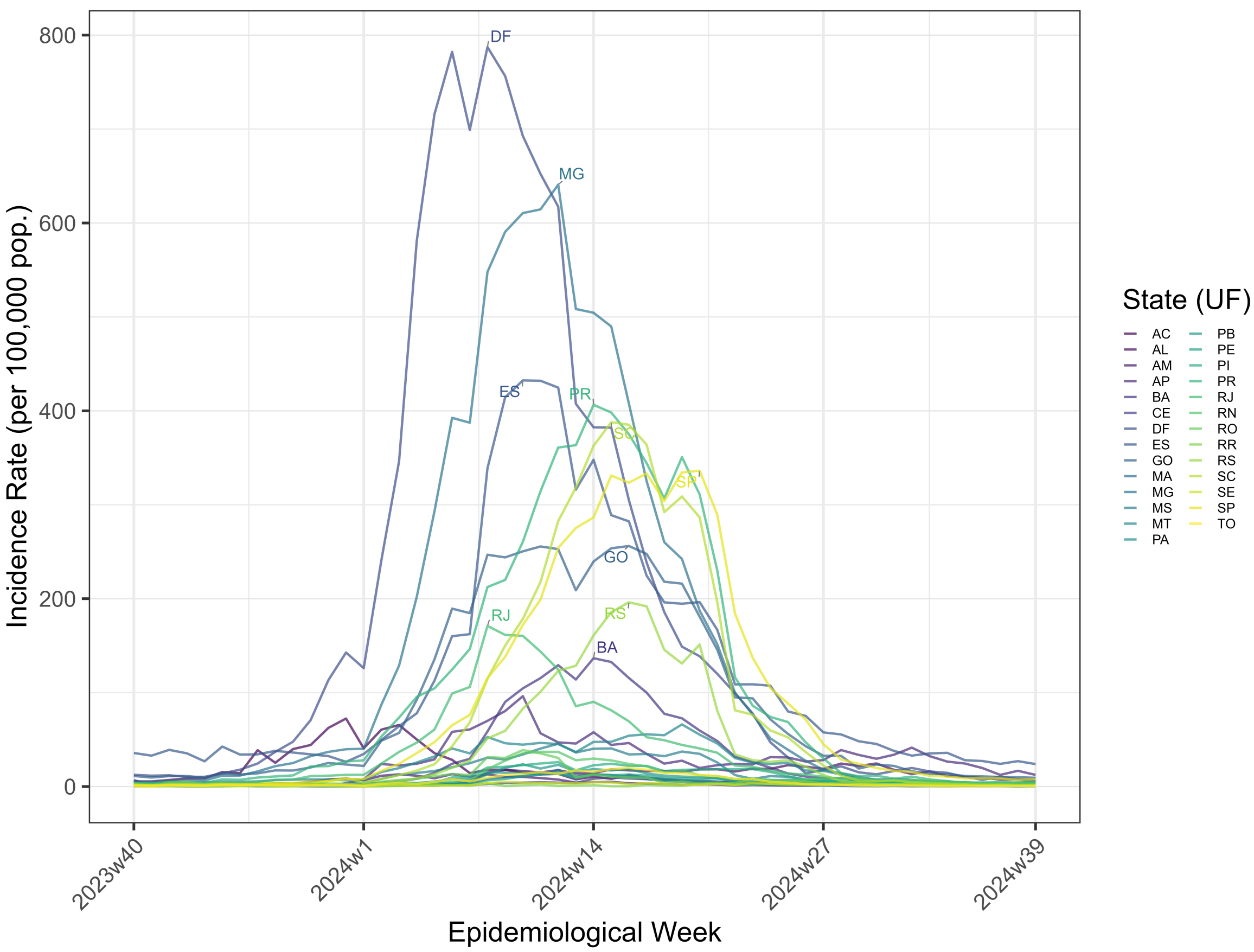

Source: Authors' elaboration based on data from the Notifiable Diseases Information System (SINAN)

A.1.1.5 Incidence of Dengue in 26 State Capitals and Brasília, 2023w40 to 2024w39

Figure 5: Incidence of Dengue in State Capitals, 2023w40 to 2024w39

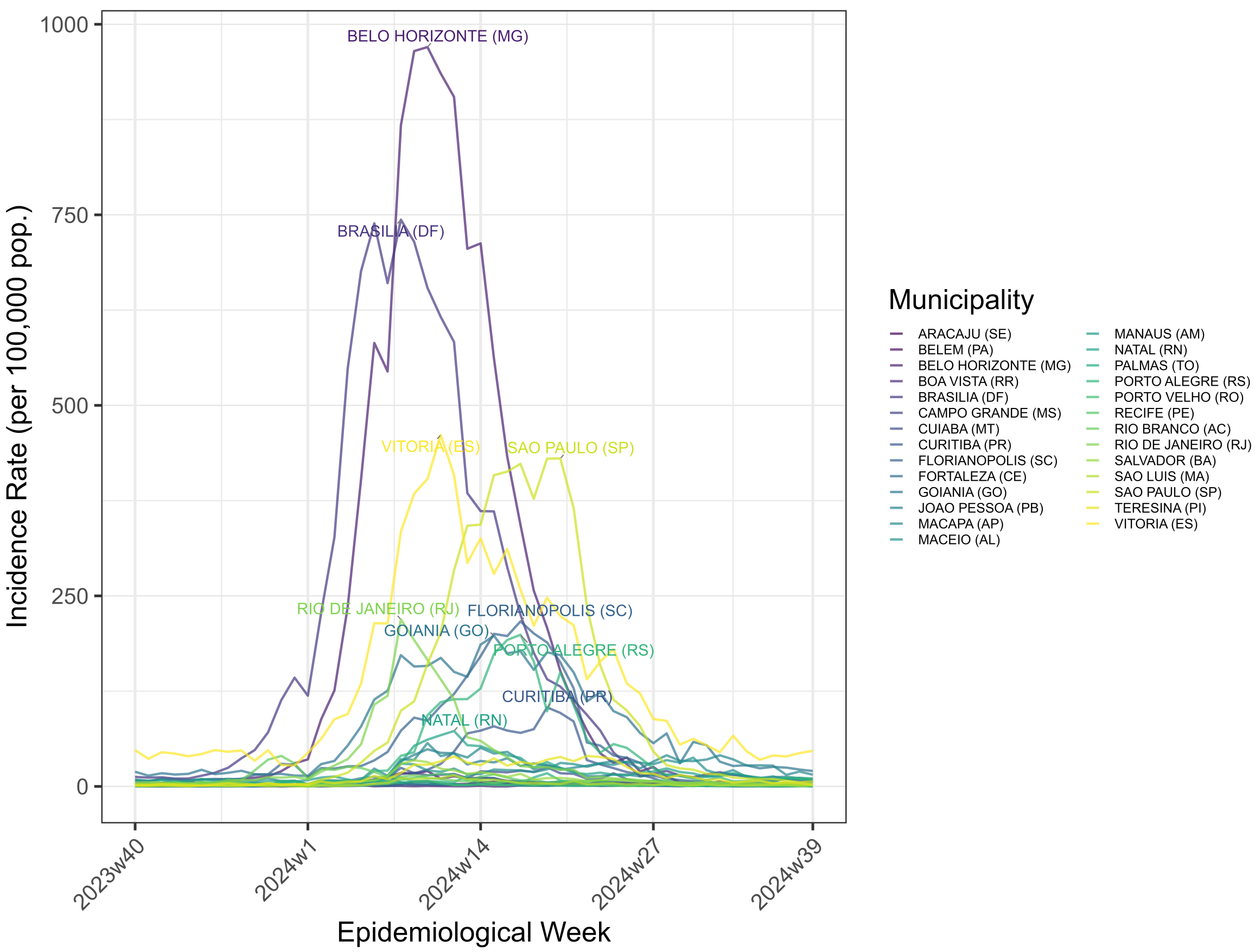

Source: Authors' elaboration based on data from the Notifiable Diseases Information System (SINAN)

A.1.1.6 Map of Cumulative Dengue Incidence, per States and Capitals, 2023w40-2024w39

Figure 6: Map of Cumulative Dengue Incidence, per States and Capitals, 2023w40-2024w39

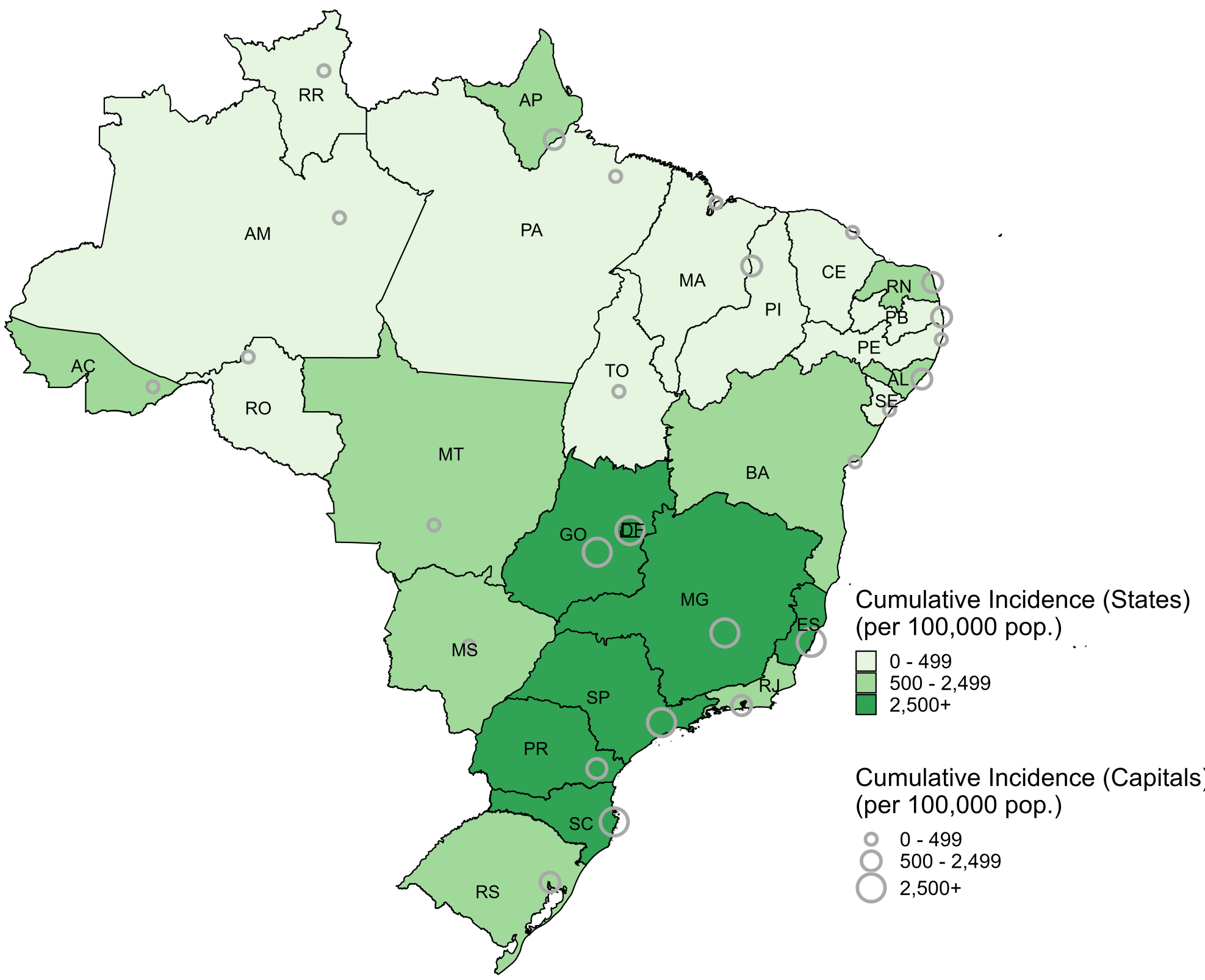

Source: Authors' elaboration based on data from the Notifiable Diseases Information System (SINAN)

A.1.1.7 Deaths Due to Dengue in Brazil, Epidemiological Years 2015-2024

Figure 7: Deaths Due to Dengue in Brazil, 2015-2024

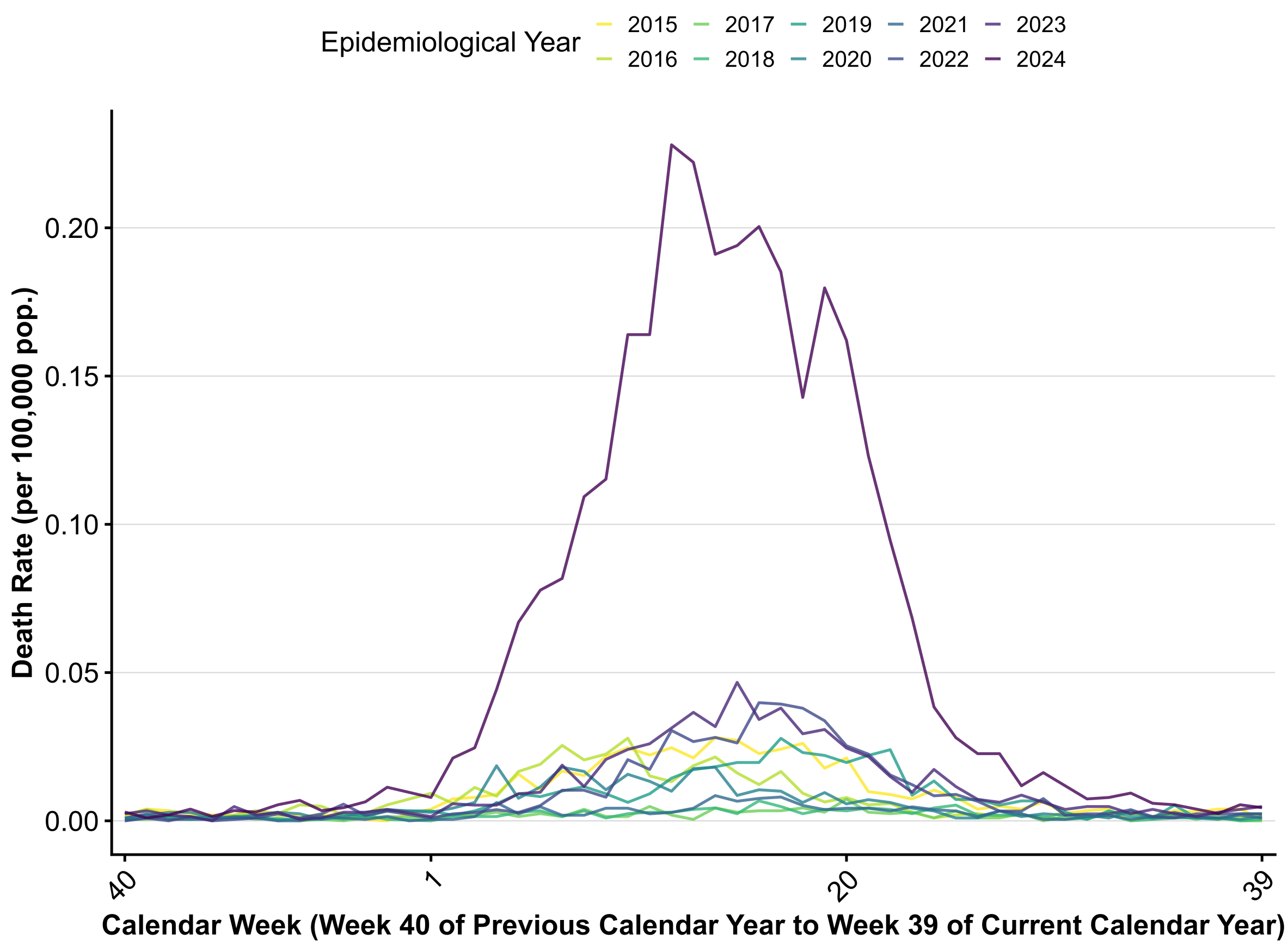

Source: Authors' elaboration based on data from the Notifiable Diseases Information System (SINAN)

A.1.1.8 Deaths per Year Due to Dengue in 26 States and the Federal District, Epidemiological Years 2015-2024

Figure 8: Deaths per Year Due to Dengue, per State, 2015-2024

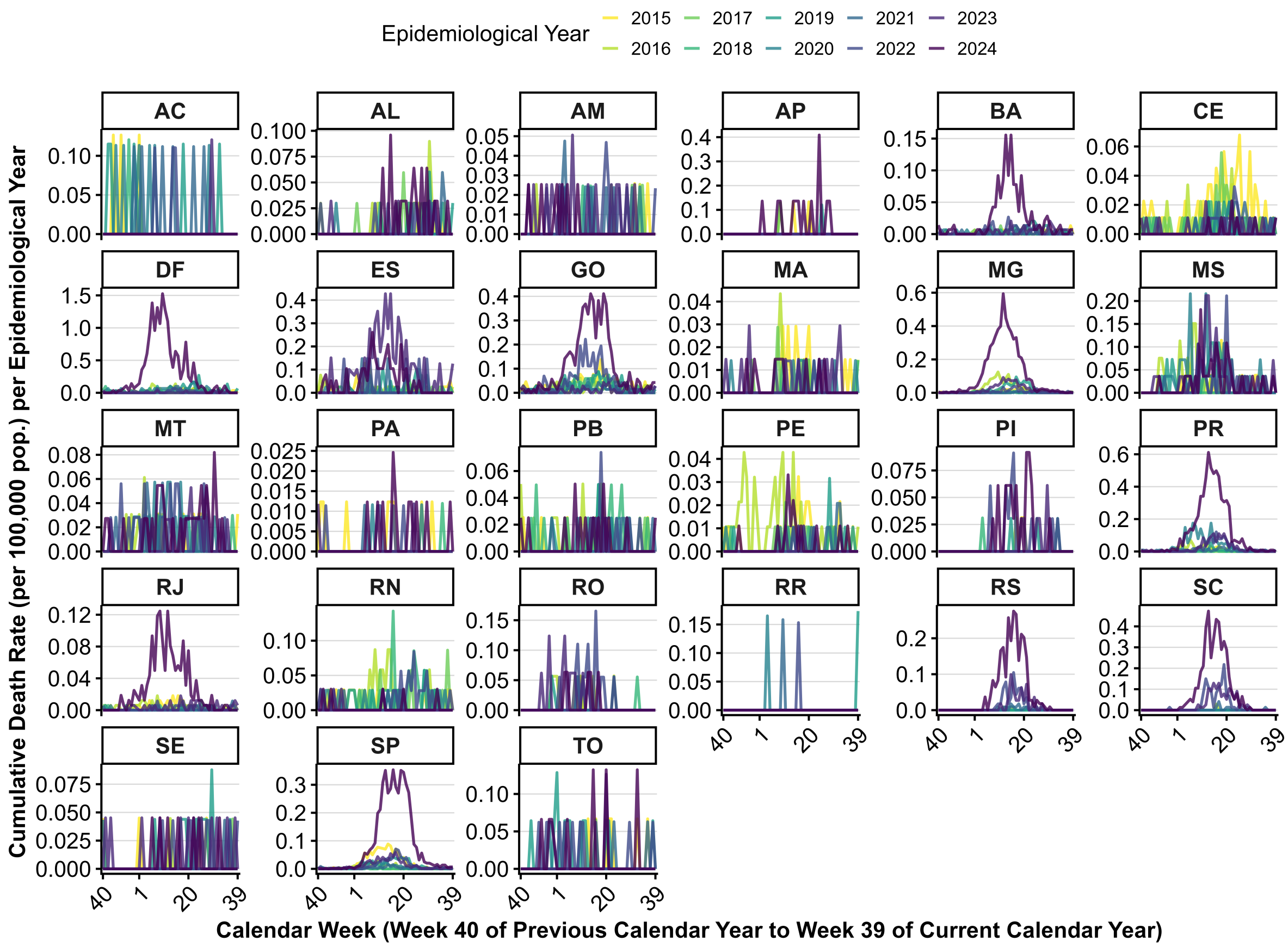

Source: Authors' elaboration based on data from the Notifiable Diseases Information System (SINAN)

A.1.1.9 Deaths per Year Due to Dengue in 26 State Capitals and Brasília, Epidemiological Years 2015-2024

Figure 9: Deaths per Year Due to Dengue in State Capitals, 2015-2024

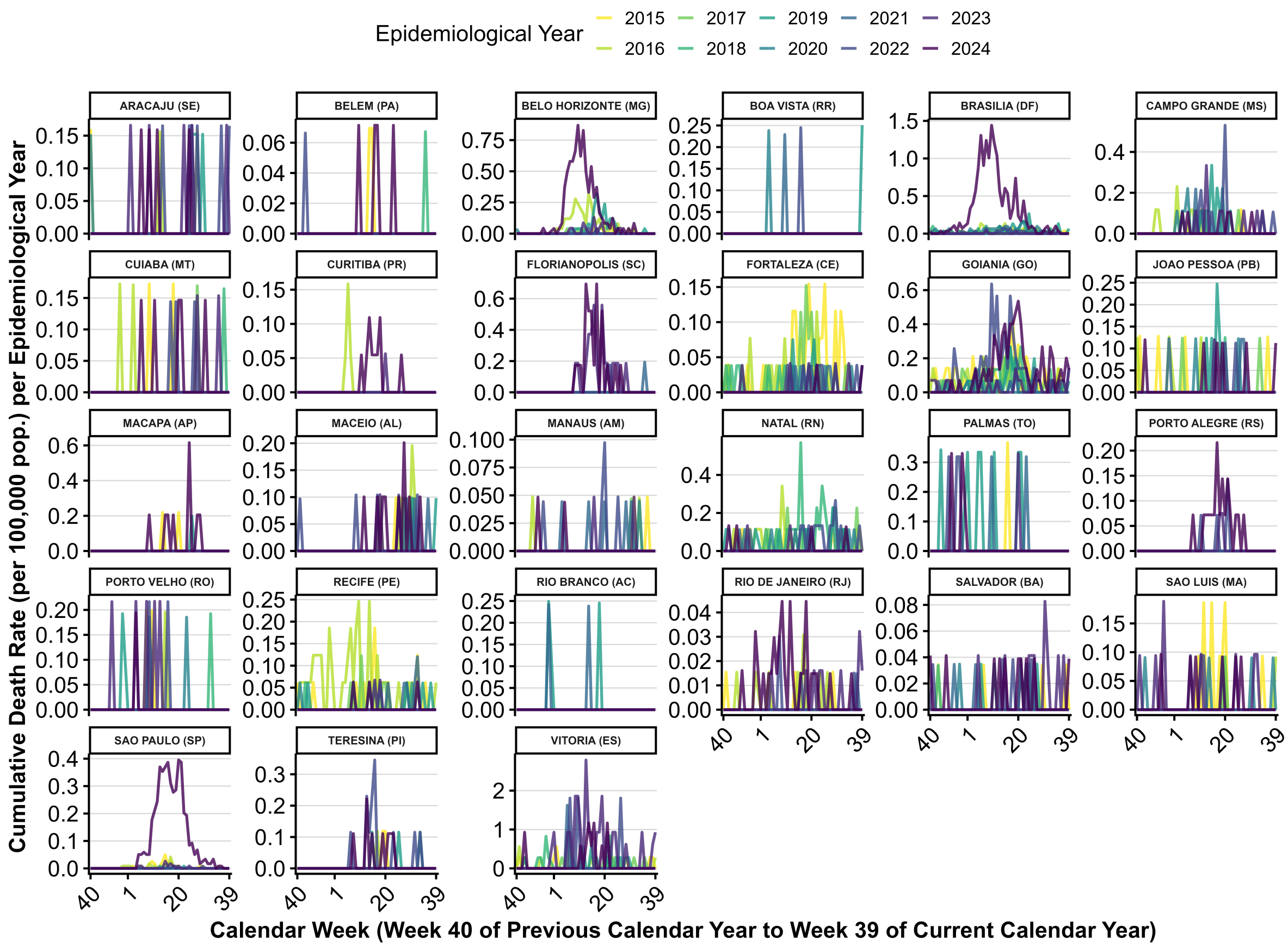

Source: Authors' elaboration based on data from the Notifiable Diseases Information System (SINAN)

A.1.1.10 Deaths Due to Dengue in 26 States and the Federal District, 2023w40 to 2024w39

Figure 10: Incidence of Dengue per State, 2023w40 to 2024w39

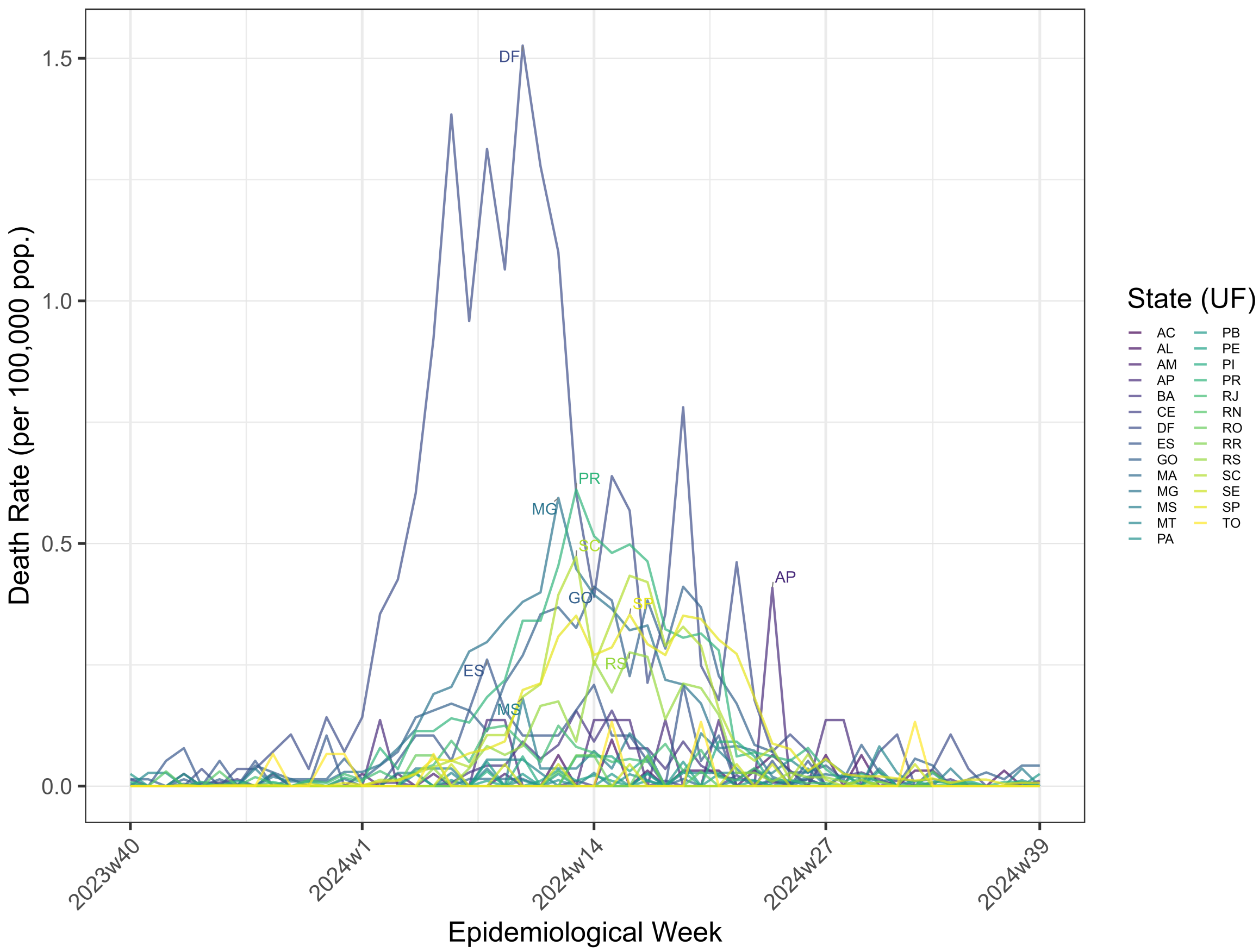

Source: Authors' elaboration based on data from the Notifiable Diseases Information System (SINAN)

A.1.1.11 Deaths Due to Dengue in 26 State Capitals and Brasília, 2023w40 to 2024w39

Figure 11: Incidence of Dengue in State Capitals, 2023w40 to 2024w39

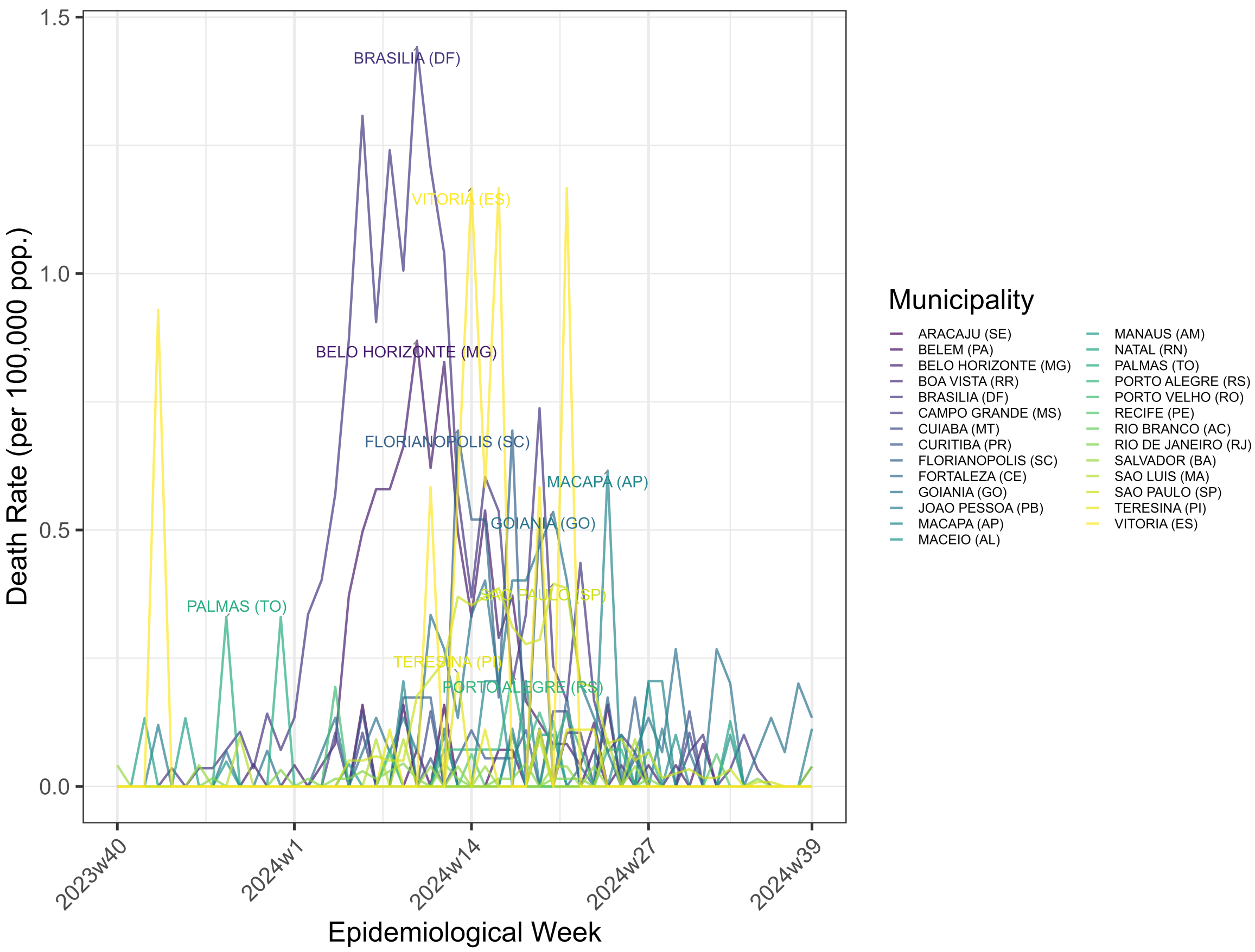

Source: Authors' elaboration based on data from the Notifiable Diseases Information System (SINAN)

A.1.1.12 Map of Cumulative Dengue Death Rate, per States and Capitals, 2023w40-2024w39

Figure 12: Map of Cumulative Dengue Death Rate, per States and Capitals, 2023w40 to 2024w39

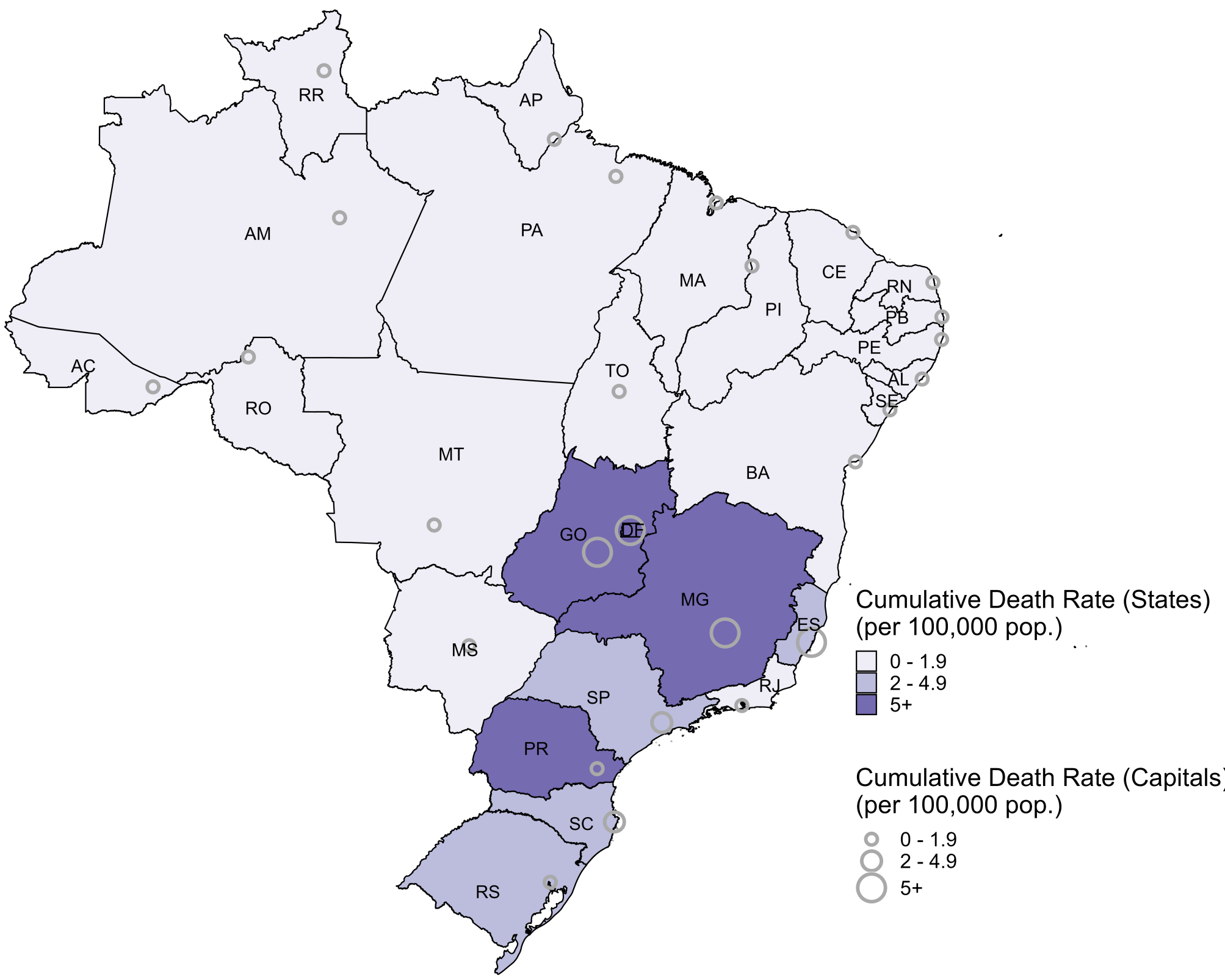

Source: Authors' elaboration based on data from the Notifiable Diseases Information System (SINAN)

A.1.2 Brazilian Arboviruses Contingency Plans, 2014-2025

Table 1: List of contingency plans and related documents by disease for federal government.

Disease Group	Disease	Title	Year	Country	Source
Arboviruses	Dengue	National Contingency Plan for Dengue Epidemics	2015	Brazil	Brazilian Virtual Health Library
Arboviruses	Dengue, Zika and Chikungunya	National contingency plan for response to public health emergencies due to dengue, chikungunya and Zika	2022	Brazil	Ministry of Health
Arboviruses	Dengue, Zika, Chikungunya and Oropouche	Guide: Action Plan for reduction of dengue and other arboviruses 2024-2025	2024	Brazil	Ministry of Health
Arboviruses	Dengue, Zika and Chikungunya	National Contingency Plan for Dengue, Chikungunya and Zika	2025	Brazil	Ministry of Health
Arboviruses	Chikungunya	National Contingency Plan for Chikungunya	2014	Brazil	Brazilian Virtual Health Library
Arboviruses	Microcephaly for Zika	National Plan for Combating Microcephaly - Health Care Protocol and Response to the Occurrence of Microcephaly	2016	Brazil	Brazilian Virtual Health Library

Source: Ministry of Health publications and Brazilian Virtual Health Library.

Contingency plans issued by the federal government were identified through manual searches of Ministry of Health publications and the Brazilian Virtual Health Library. As a robustness check, freedom of information requests were submitted to the federal government to confirm whether additional contingency plans had been issued.

Table 2: List of contingency plans and related documents by disease for state governments.

Disease Group	Disease	Title	Year	State	Source
Arboviruses	Dengue, Zika and Chikungunya	State Contingency Plan for Combating Arboviral Diseases	2024/2026	Amapá	State Department of Health
Arboviruses	Dengue	Preparedness, Surveillance and Response Plan for Dengue, Zika and Chikungunya in Amazonas 2023/2024	2023/2024	Amazonas	Amazonas Health Surveillance Foundation
Arboviruses	Dengue, Zika and Chikungunya	Integrated State Health Plan: Confrontation of Arboviruses 2024/2025	2024/2025	Ceará	State Department of Health

Continued on next page

Table 2: **List of contingency plans and related documents by disease for state governments.** (Continued)

Arboviruses	Dengue, Zika and Chikungunya	Contingency Plan for responding to public health emergencies caused by Dengue, Chikungunya, and Zika	2024/2027	Distrito Federal	State Department of Health
Arboviruses	Dengue, Zika and Chikungunya	State Contingency Plan for Arboviral Diseases: Dengue, Zika, and Chikungunya	2021/2023	Espírito Santo	State Department of Health
Arboviruses	Dengue, Zika and Chikungunya	State Contingency Plan for Arboviral Diseases	2024/2026	Espírito Santo	State Department of Health
Arboviruses	Dengue, Zika and Chikungunya	Contingency Plan for Arbovirus Control	2015/2016	Goiás	State Department of Health
Arboviruses	Dengue, Zika and Chikungunya	Contingency Plan for Arbovirus Control	2023/2024	Goiás	State Department of Health
Arboviruses	Dengue, Zika and Chikungunya	Contingency Plan for Arbovirus Control	2025/2026	Goiás	State Department of Health
Arboviruses	Dengue, Zika and Chikungunya	Contingency Plan for the prevention and control of arbovirus epidemics in the State of Maranhão: Dengue, Chikungunya and Zika	2022/2023	Maranhão	State Department of Health
Arboviruses	Dengue, Zika and Chikungunya	Contingency Plan for the prevention and control of arbovirus epidemics in the State of Maranhão: Dengue, Chikungunya and Zika	2023/2024	Maranhão	State Department of Health
Arboviruses	Dengue, Zika and Chikungunya	Contingency Plan for the prevention and control of arbovirus epidemics in the State of Maranhão: Dengue, Chikungunya and Zika	2024/2025	Maranhão	State Department of Health
Arboviruses	Dengue, Zika and Chikungunya	Contingency Plan for Urban Arboviruses Dengue, Chikungunya, Zika and in the State of Mato Grosso	2023/2024	Mato Grosso	State Department of Health
Arboviruses	Dengue, Zika and Chikungunya	Contingency Plan for Confronting Dengue, Zika and Chikungunya	2025/2026	Mato Grosso	State Department of Health
Arboviruses	Dengue, Zika and Chikungunya	State Contingency Plan for Arboviruses: Dengue, Zika, and Chikungunya	2022	Paraíba	State Department of Health
Arboviruses	Dengue, Zika and Chikungunya	State Contingency and Control Plan for Arboviruses: Dengue, Zika, Chikungunya and Oropouche	2025/2026	Paraíba	State Department of Health
Arboviruses	Arbovirus	Arbovirus Confronting Plan	2020	Pernambuco	State Department of Health
Arboviruses	Arbovirus	Arbovirus Confronting Plan	2024	Pernambuco	State Department of Health
Arboviruses	Arbovirus	Arbovirus Confronting Plan	2025/2026	Pernambuco	State Department of Health

Continued on next page

Table 2: **List of contingency plans and related documents by disease for state governments.** (Continued)

Arboviruses	Dengue	Contingency Plan for Confronting Dengue	2013/2015	Piauí	State Department of Health
Arboviruses	Dengue, Zika and Chikungunya	Contingency Plan for Confronting Arboviruses Transmitted by Aedes Aegypti	2018/2019	Rio de Janeiro	State Department of Health
Arboviruses	Dengue, Zika and Chikungunya	State Contingency Plan for responding to public health emergencies caused by Dengue, Chikungunya, and Zika	2022	Rio Grande do Norte	State Department of Health
Arboviruses	Arbovirus	State Contingency Plan for Arbovirus Infection	2022/2025	Rio Grande do Norte	State Department of Health
Arboviruses	Dengue, Zika and Chikungunya	Contingency Plan for responding to public health emergencies caused by Dengue, Chikungunya, and Zika	2025/2026	Rio Grande do Norte	State Department of Health
Arboviruses	Dengue	Contingency Plan for Dengue	2008	Rio Grande do Sul	State Department of Health
Arboviruses	Dengue	Contingency Plan for Dengue	2011	Rio Grande do Sul	State Department of Health
Arboviruses	Dengue	Contingency Plan for Dengue	2013	Rio Grande do Sul	State Department of Health
Arboviruses	Dengue, Zika and Chikungunya	Integrated Contingency Plan for Dengue, Chikungunya and Zika Virus	2015	Rio Grande do Sul	State Department of Health
Arboviruses	Dengue	State Plan for Integrated Actions for Health Surveillance and Primary Care for arboviruses with a focus on dengue	2021	Rio Grande do Sul	State Department of Health
Arboviruses	Dengue, Zika and Chikungunya	Contingency Plan for confronting Urban Arboviruses: Dengue, Zika, and Chikungunya	2022/2023	Rio Grande do Sul	State Department of Health
Arboviruses	Dengue	Contingency Plan for confronting Dengue	2024/2025	Rio Grande do Sul	State Department of Health
Arboviruses	Dengue, Zika and Chikungunya	Contingency Plan for Dengue, Zika, and Chikungunya	2017/2018	Rondônia	State Department of Health
Arboviruses	Dengue	Contingency Plan for Confronting Dengue in the State of Santa Catarina	2015	Santa Catarina	State Department of Health
Arboviruses	Dengue, Zika and Chikungunya	Contingency Plan for Confronting Dengue, Chikungunya Fever and Zika Virus in the State of Santa Catarina	2017	Santa Catarina	State Department of Health

Continued on next page

Table 2: **List of contingency plans and related documents by disease for state governments.** (Continued)

Arboviruses	Dengue, Zika and Chikungunya	Contingency Plan for Confronting Dengue, Chikungunya Fever and Zika Virus in the State of Santa Catarina	2019	Santa Catarina	State Department of Health
Arboviruses	Dengue, Zika and Chikungunya	Contingency Plan for Confronting Dengue, Chikungunya Fever and Zika Virus in the State of Santa Catarina	2021	Santa Catarina	State Department of Health
Arboviruses	Dengue, Zika and Chikungunya	Contingency Plan to Combat Dengue, Chikungunya and Zika in the State of Santa Catarina	2022	Santa Catarina	State Department of Health
Arboviruses	Dengue, Zika and Chikungunya	Contingency Plan to Combat Dengue, Chikungunya and Zika in the State of Santa Catarina	2023	Santa Catarina	State Department of Health
Arboviruses	Dengue, Zika and Chikungunya	Contingency Plan for Confronting Dengue, Chikungunya and Zika in the State of Santa Catarina	2024	Santa Catarina	State Department of Health
Arboviruses	Dengue	Contingency plan for dengue in the State of São Paulo	2015/2016	São Paulo	State Department of Health
Arboviruses	Dengue, Zika and Chikungunya	Contingency plan for arboviruses in the State of São Paulo	2017	São Paulo	State Department of Health
Arboviruses	Dengue, Zika and Chikungunya	State contingency plan against urban arboviruses	2018	São Paulo	State Department of Health
Arboviruses	Dengue, Zika and Chikungunya	State Contingency Plan for Urban Arboviruses: Dengue, Chikungunya and Zika 2023-2024	2023/2024	São Paulo	State Department of Health
Arboviruses	Dengue, Zika and Chikungunya	State contingency plan for urban arboviruses: Dengue, Zika and Chikungunya	2025/2026	São Paulo	State Department of Health
Arboviruses	Dengue, Zika and Chikungunya	Contingency Plan for responding to Dengue, Chikungunya, and Zika epidemics	2024/2025	Sergipe	State Department of Health
Arboviruses	Dengue	Plan for the Prevention and Control of Dengue Epidemics in Tocantins	2010/2011	Tocantins	State Department of Health
Arboviruses	Dengue	Strategies for the Prevention and Control of Dengue Epidemics in Tocantins (Contingency Plan)	2014/2015	Tocantins	State Department of Health
Arboviruses	Dengue, Zika and Chikungunya	State Contingency Plan for Arboviral Diseases in Tocantins	2021	Tocantins	State Department of Health

Continued on next page

Table 2: **List of contingency plans and related documents by disease for state governments.** (Continued)

Arboviruses	Dengue, Zika and Chikungunya	State Contingency Plan for the Prevention and Control of Arboviral Diseases	2024/2025	Tocantins	State Department of Health

Source: Authors' elaboration from state health department documents.

Contingency plans were obtained in two ways. For the initial sample of states—Rio de Janeiro, São Paulo, Santa Catarina, Mato Grosso, Amazonas and Ceará—documents were collected manually and also requested through the Brazilian Access to Information Law (Lei de Acesso à Informação, LAI). For all other states, contingency plans were requested exclusively through LAI. Four states did not respond to the LAI requests: Acre, Roraima, Paraná and Pará. Therefore, contingency plans for these states are not listed in this table. Other states not listed here did not provide any contingency plan document.

Table 3: **List of contingency plans and related documents by disease for municipal governments.**

**Disease Group**	**Disease**	**Title**	**Year**	**City**	**Source**
Arboviruses	Dengue	Contingency Plan for assisting dengue patients in Aracaju	2010	Aracaju	Municipal Health Department
Arboviruses	Dengue	Contingency Plan for assisting dengue patients in Aracaju	2011	Aracaju	Municipal Health Department
Arboviruses	Dengue, Zika and Chikungunya	Contingency Plan for assisting Dengue, Chikungunya, and Zika patients in Aracaju	2024	Aracaju	Municipal Health Department
Arboviruses	Dengue, Zika and Chikungunya	Contingency Plan for addressing the care of patients with dengue, chikungunya, and Zika in Aracaju	2024	Aracaju	Municipal Health Department
Arboviruses	Dengue, Zika and Chikungunya	Municipal Contingency Plan for confronting Dengue, Chikungunya and Zika	2015/2016	Belo Horizonte	Municipal Health Department
Arboviruses	Dengue	Contingency plan for disasters resulting from Aedes Aegypti infestation.	2016	Belo Horizonte	Municipal Health Department
Arboviruses	Dengue, Chikungunya and Zika	Contingency plan for confronting arboviruses transmitted by Aedes aegypti.	2021	Belo Horizonte	Municipal Health Department
Arboviruses	Dengue, Chikungunya and Zika	Contingency plan for confronting arboviruses transmitted by Aedes aegypti.	2023	Belo Horizonte	Municipal Health Department
Arboviruses	Dengue, Chikungunya and Zika	Contingency plan for confronting arboviruses transmitted by Aedes aegypti.	2024/2025	Belo Horizonte	Municipal Health Department
Arboviruses	Dengue and chikungunya	Municipal Contingency Plan for Confronting Dengue and Chikungunya 2015/2016	2015-2016	Campinas	Municipal Health Department

Continued on next page

Table 3: **List of contingency plans and related documents by disease for municipal governments.** (Continued)

Arboviruses	Dengue and chikungunya	Municipal Contingency Plan to Combat Arboviruses	2017/2018	Campinas	Municipal Health Department
Arboviruses	Dengue and chikungunya	Municipal Contingency Plan for Combating Urban Arboviruses	2019/2020	Campinas	Municipal Health Department
Arboviruses	Dengue and chikungunya	Municipal Contingency Plan to Combat Combating Urban Arboviruses	2021/2022	Campinas	Municipal Health Department
Arboviruses	Dengue and chikungunya	Municipal Contingency Plan to Combat Urban Arboviruses 2023/2024	2023/2024	Campinas	Municipal Health Department
Arboviruses	Dengue and chikungunya	Public Health Emergency Response Plan for Urban Arboviruses	2024	Campinas	Municipal Health Department
Arboviruses	Dengue, Chikungunya and Zika	Municipal Contingency Plan for Combating Arboviruses. Year 2025/2026	2025/2026	Campinas	Municipal Committee for Combating Arboviruses and Zoonoses
Arboviruses	Dengue, Febre chikungunya, Zika vírus	Municipal Contingency Plan for Arboviruses	2025	Cuiabá	Municipal Health Department
Arboviruses	Dengue, Febre chikungunya, Zika vírus	Plan to Combat Dengue and Other Arboviruses in the Municipality of Florianópolis	2024	Florianópolis	Municipal Health Department
Arboviruses	Dengue, Febre chikungunya, Zika vírus	Plan to Combat Dengue and Other Arboviruses in the Municipality of Florianópolis	2025	Florianópolis	Municipal Health Department
Arboviruses	Dengue, Chikungunya and Zika	Contingency Plan for confronting and controlling arbovirus epidemics	2018	Fortaleza	Municipal Health Department
Arboviruses	Dengue, Chikungunya and Zika	Contingency Plan for Confronting and Controlling Epidemics by Arboviruses	2021	Fortaleza	Municipal Health Department
Arboviruses	Dengue, Zika and Chikungunya	Contingency Plan for Dengue, Zika, and Chikungunya	2018	Maceió	Municipal Health Department
Arboviruses	Dengue, Zika and Chikungunya	Contingency Plan for Dengue, Zika, and Chikungunya	2020	Maceió	Municipal Health Department
Arboviruses	Dengue, Zika and Chikungunya	Contingency Plan for Dengue, Zika, and Chikungunya	2022	Maceió	Municipal Health Department
Arboviruses	Dengue, Zika and Chikungunya	Contingency Plan for Dengue, Zika, and Chikungunya	2023/2025	Maceió	Municipal Health Department
Arboviruses	Dengue	Contingency Plan for Surveillance and Control of Dengue in Manaus - 2014 to 2017	2014/2017	Manaus	Municipal Health Department
Arboviruses	Dengue, Chikungunya and Zika	Arbovirus Contingency Plan Manaus 2024	2024	Manaus	Municipal Health Department

Continued on next page

Table 3: **List of contingency plans and related documents by disease for municipal governments.** (Continued)

Arboviruses	Dengue, Chikungunya, Zika, Oropouche and Mayaro	Arbovirus Contingency Plan	2025	Manaus	Municipal Health Department
Arboviruses	Dengue, Zika and Chikungunya	Contingency Plan for the prevention and control of arboviruses in the municipality of Palmas, Tocantins	2024/2025	Palmas	Municipal Health Department
Arboviruses	Dengue	Contingency Plan for Confronting Dengue	2010	Porto Alegre	Municipal Health Department
Arboviruses	Dengue	Contingency Plan for Confronting Dengue	2013	Porto Alegre	Municipal Health Department
Arboviruses	Dengue	Contingency Plan for Confronting Dengue	2015	Porto Alegre	Municipal Health Department
Arboviruses	Dengue, Zika and Chikungunya	Contingency Plan for Confronting Dengue, Zika and Chikunguya	2016	Porto Alegre	Municipal Health Department
Arboviruses	Dengue, Zika and Chikungunya	Contingency Plan for Confronting Dengue, Zika and Chikunguya	2019	Porto Alegre	Municipal Health Department
Arboviruses	Dengue, Zika and Chikungunya	Contingency Plan for Confronting Dengue, Zika and Chikunguya	2022	Porto Alegre	Municipal Health Department
Arboviruses	Dengue, Zika and Chikungunya	Contingency Plan for Confronting Dengue, Zika and Chikunguya	2023	Porto Alegre	Municipal Health Department
Arboviruses	Dengue, Zika and Chikungunya	Contingency Plan for Confronting Dengue, Zika and Chikunguya	2024	Porto Alegre	Municipal Health Department
Arboviruses	Dengue, Zika and Chikungunya	Contingency Plan for Confronting Dengue, Zika and Chikunguya	2025	Porto Alegre	Municipal Health Department
Arboviruses	Dengue, Chikunguya, Zika and Oroupuche	Municipal Contingency Plan for Addressing Arboviruses (Dengue, Zika Virus, Chikungunya Fever, and Oropouche Sickness)	2024/2025	Porto Velho	Municipal Health Department
Arboviruses	Dengue, Chikunguya, Zika and Oroupuche	Emergency Plan for responding to public health emergencies caused by Dengue, Chikungunya, Zika, and Oropouche	2025/2026	Recife	Municipal Health Department
Arboviruses	Dengue, Zika and Chikungunya	Contingency Plan for Confronting Arbovirus (Dengue, Zika and Chikunguya)	2023	Rio Branco	Municipal Health Department
Arboviruses	Dengue, Chikungunya and Zika	Contingency Plan for Confronting Arbovirus	2024/2025	Rio Branco	Municipal Health Department

Continued on next page

Table 3: **List of contingency plans and related documents by disease for municipal governments.** (Continued)

Arboviruses	Dengue, Febre chikungunya, Zika vírus	Municipal Contingency Plan for Arboviruses: Dengue, Chikungunya and Zika (2024-2025)	2024	Rio de Janeiro	Municipal Health Department
Arboviruses	Dengue, Febre chikungunya, Zika vírus	Municipal Contingency Plan for Dengue, Chikungunya and Zika 2021-2023	2021/2023	Rio de Janeiro	Municipal Health Department
Arboviruses	Dengue, Zika and Chikungunya	Municipal contingency plan for urban arboviruses: Dengue, Zika and Chikungunya	2024/2025	Rio de Janeiro	Municipal Health Department
Arboviruses	Dengue, Zika and Chikungunya	Municipal plan to combat dengue and other arboviruses	2022/2023	São Paulo	Municipal Health Department
Arboviruses	Dengue, Zika and Chikungunya	Municipal plan to combat dengue and other arboviruses	2023/2024	São Paulo	Municipal Health Department
Arboviruses	Dengue	Contingency Plan for Dengue Control and Patient Care	2010/2011	Vitória	Municipal Health Department
Arboviruses	Dengue	Contingency Plan for Dengue Control and Patient Care	2012/2013	Vitória	Municipal Health Department
Arboviruses	Dengue	Contingency Plan for Dengue Control and Patient Care	2013/2014	Vitória	Municipal Health Department
Arboviruses	Dengue	Contingency Plan for Dengue Control and Patient Care	2015/2016	Vitória	Municipal Health Department
Arboviruses	Dengue, Zika and Chikungunya	Contingency Plan for the control of arboviruses (Dengue, Zika and Chikungunya) and patient care.	2022/2023	Vitória	Municipal Health Department
Arboviruses	Dengue, Zika and Chikungunya	Contingency Plan for the control of arboviruses (Dengue, Zika and Chikungunya) and patient care.	2024/2025	Vitória	Municipal Health Department

Source: Authors' elaboration from municipal health department documents.

Contingency plans were obtained in two ways. For the initial sample of capitals/city—Rio de Janeiro, Campinas (city), Florianópolis, Cuiabá, Manaus and Fortaleza—documents were collected manually and also requested through the Brazilian Access to Information Law (Lei de Acesso à Informação, LAI). For all other capitals, contingency plans were requested exclusively through LAI. Six capitals did not respond to the LAI requests: Boa Vista, Salvador, Macapá, Belém, Natal and São Luís. Therefore, contingency plans for these capitals are not listed in this table. Other capitals not listed here did not provide any contingency plan document.

Table 4: **Risk level classification and endemic channel adoption in the most recent contingency plans from state and municipal governments**

Risk Classification Analysis						
**Federal Unit**	**City**	**Federal Entity**	**Most Recent Year**	**Risk Level**	**Number of Risk Level**	**Endemic Channel**
Acre	Rio Branco	Municipal	2024/2025	Yes	3 levels	Yes
Alagoas	Maceió	Municipal	2023/2025	Yes	3 levels	Yes
Amazonas	Manaus	Municipal	2025	Yes	3 levels	Yes
Ceará	Fortaleza	Municipal	2021	No	-	Yes
Espírito Santo	Vitória	Municipal	2024/2025	Yes	4 levels	Yes
Mato Grosso	Cuiabá	Municipal	2025	Yes	4 levels	Yes
Minas Gerais	Belo Horizonte	Municipal	2024/2025	Yes	5 levels	No
Pernambuco	Recife	Municipal	2025/2026	Yes	3 levels	Yes
Rio de Janeiro	Rio de Janeiro	Municipal	2024/2025	Yes	4 levels	Yes
Rio Grande do Sul	Porto Alegre	Municipal	2025	Yes	5 levels	Yes
Rondônia	Porto Velho	Municipal	2024/2025	Yes	3 levels	Yes
São Paulo	Campinas	Municipal	2025/2026	No	-	Yes
São Paulo	São Paulo	Municipal	2023/2024	Yes	4 levels	No
Santa Catarina	Florianópolis	Municipal	2025	Yes	4 levels	Yes
Sergipe	Aracaju	Municipal	2024	Yes	4 levels	Yes
Tocantins	Palmas	Municipal	2024/2025	Yes	4 levels	Yes
Amapá	-	State	2024/2026	Yes	4 levels	Yes
Amazonas	-	State	2023/2024	Yes	4 levels	No
Ceará	-	State	2024/2025	Yes	5 levels	Yes
Distrito Federal	-	UF	2024/2027	Yes	4 levels	Yes
Espírito Santo	-	State	2024/2026	Yes	4 levels	Yes
Goiás	-	State	2025/2026	Yes	3 levels	Yes
Maranhão	-	State	2024/2025	No	-	No
Mato Grosso	-	State	2025/2026	Yes	4 levels	Yes
Paraíba	-	State	2025/2026	Yes	4 levels	Yes
Pernambuco	-	State	2025/2026	Yes	3 levels	Yes
Piauí	-	State	2013/2015	No	-	No
Rio de Janeiro	-	State	2018/2019	Yes	3 levels	No
Rio Grande do Norte	-	State	2025/2026	Yes	3 levels	Yes

Continued on next page

Table 4: **Risk level classification and endemic channel adoption in the most recent contingency plans from state and municipal governments**

(Continued)

Rio Grande do Sul	-	State	2024/2025	Yes	5 levels	Yes
Rondônia	-	State	2017/2018	No	-	No
Santa Catarina	-	State	2024	Yes	3 levels	No
São Paulo	-	State	2025/2026	Yes	3 levels	Yes
Sergipe	-	State	2024/2025	Yes	3 levels	Yes
Tocantins	-	State	2024/2025	Yes	4 levels	Yes

Source: Authors' elaboration from state and municipal health department documents.

Contingency plans were obtained in two ways. For the initial sample of states and capitals, documents were collected manually and also requested through the Brazilian Access to Information Law (Lei de Acesso à Informação, LAI). For all other locations, contingency plans were requested exclusively through LAI. Four states (Acre, Roraima, Paraná and Pará) and six capitals (Boa Vista, Salvador, Macapá, Belém, Natal and São Luís) did not respond to the LAI requests. Therefore, locations that did not respond or did not submit any contingency plan are not listed in this table. Only the most recent contingency plan submitted by each government is included.

A.1.3 Public Health Emergency Declarations

A.1.3.1 Sources and List of terms used to search for health emergency declarations

Sources: All sites with the following domains: ".am.gov.br", ".ce.gov.br", ".ceara.gov.br", ".rj.gov.br", ".rio.gov.br", ".sc.gov.br", ".mt.gov.br", and ".sp.gov.br". The public custom search engine can be accessed through the following address: https://cse.google.com/cse?cx=000e89324352d4c64

List of terms: "declaração de emergência sanitária" | "declaração de emergência em saúde" | "declaração de emergência em função de evento inusitado em saúde" | "declaração de emergência por doença infecciosa" | "declaração de emergência por doença epidêmica" | "declaração de emergência por epidemia" | "declaração de situação de emergência" | "declaração de regime de emergência em saúde" | "declaração de gravidade de situação epidemiológica" | "situação de emergência epidemiológica" | "declaração de situação de emergência epidemiológica" | "Reconhecimento de situação de emergência epidemiológica" | "declaração de perigo à saúde pública" | "declaração de situação anormal em saúde" | "declaração de situação de alerta em saúde" | "declaração de alerta em saúde" | "declaração de crise sanitária" | "declaração de situação de crise sanitária" | "declaração de situação excepcionalidade em saúde" | "declaração de situação calamidade pública" | "declaração de situação calamidade sanitária" | "declaração de estado de calamidade pública" | "declaração de estado de calamidade sanitária" | "declaração de emergência em saúde pública" | "declaração de emergência de saúde pública" | "reconhecimento de emergência sanitária" | "reconhecimento de emergência em saúde" | "reconhecimento de emergência em função de evento inusitado em saúde" | "reconhecimento de emergência por doença infecciosa" | "reconhecimento de emergência por doença epidêmica" | "reconhecimento de emergência por epidemia" | "reconhecimento de situação de emergência" | "reconhecimento de regime de emergência em saúde" | "reconhecimento de gravidade da situação epidemiológica" | "reconhecimento de situação de emergência epidemiológica" | "Reconhecimento de situação de emergência epidemiológica" | "reconhecimento de perigo à saúde pública" | "reconhecimento de situação anormal em saúde" | "reconhecimento de situação de alerta em saúde" | "reconhecimento de alerta em saúde" | "reconhecimento de crise sanitária" | "reconhecimento de situação de crise sanitária" | "reconhecimento de situação excepcionalidade em saúde" | "reconhecimento de situação calamidade pública" | "reconhecimento de situação calamidade sanitária" | "reconhecimento de estado de calamidade pública" | "reconhecimento de estado de calamidade sanitária" | "reconhecimento de emergência em saúde pública" | "reconhecimento de emergência de saúde pública" | "decreto de emergência sanitária" | "decreto de emergência em saúde" | "decreto de emergência em função de evento inusitado em saúde" | "decreto de emergência por doença infecciosa" | "decreto de emergência por doença epidêmica" | "decreto de emergência por epidemia" | "decreto de situação de emergência" | "decreto de regime de emergência em saúde" | "decreto de gravidade de situação epidemiológica" | "decreto de situação de emergência epidemiológica" | "decreto de situação de emergência epidemiológica" | "decreto de perigo à saúde pública" | "decreto

de situação anormal em saúde" | "decreto de situação de alerta em saúde" | "decreto de alerta em saúde" | "decreto de crise sanitária" | "decreto de situação de crise sanitária" | "decreto de situação excepcionalidade em saúde" | "decreto de situação calamidade pública" | "decreto de situação calamidade sanitária" | "decreto de estado de calamidade pública" | "decreto de estado de calamidade sanitária" | "decreto de emergência em saúde pública" | "decreto de emergência de saúde pública" | "Arboviroses" | "arbovirose" | "arbovírus" | "dengue" | "febre da dengue" | "dengue clássica" | "dengue grave" | "dengue hemorrágica" | "DENV" | "zika" | "febre" | "zika" | "ZIKV" | "CHIKV" | "Febre Chikungunya" | "Chikungunya | "Febre do Oropouche" | "OROV" | "Oropouche" | "Febre amarela" | "Arbovirose hemorrágica" | "YFV" | "Flavivírus" | "Paludismo" | "Plasmodium" | "mosquitos" | "hemoparasitária"

A.1.3.2 Country Level

At the federal level, the only announcements that were identified were the creation of an emergency operations center to monitor the outbreak. With the exception of Portaria GM/MS Nº 3.140, de 2 de Fevereiro de 2024 (2024w5) which announced the creation of a situation room for a public health emergency for dengue and other arboviruses, no additional emergency declaration warnings were issued.

A.1.3.3 State Level

The table shows that 11 states issued a public health emergency declaration between 2023w40 and 2024w39.

Table 5: **State-wide Dengue-Related Emergency Declarations (chronological order)**

States	Type of Decree	Date Issued	Disease	Number	Duration (days)
Acre	Emergency	2024-01-05 (2024w1)	Arboviruses	11396/2024	90
Distrito Federal	Emergency	2024-01-25 (2024w4)	Arboviruses	45448/2024	239
Goiás	Emergency	2024-02-02 (2024w5)	Arboviruses	10405/2024	180
Espírito Santo	Emergency	2024-02-21 (2024w8)	Arboviruses (Dengue)	5623/2024	180
Rio de Janeiro	Emergency	2024-02-21 (2024w8)	Arboviruses (Dengue)	48969/2024	110
Santa Catarina	Emergency	2024-02-22 (2024w8)	Arboviruses (Dengue)	478/2024	180
Minas Gerais	Emergency	2024-02-26 (2024w9)	Arboviruses	64/2024	180
Amapá	Emergency	2024-02-28 (2024w9)	Arboviruses	1837/2024	180
São Paulo	Emergency	2024-03-05 (2024w10)	Arboviruses (Dengue)	68368/2024	202
Rio Grande do Sul	Emergency	2024-03-12 (2024w11)	Arboviruses (Dengue)	57498/2024	180
Paraná	Emergency	2024-03-14 (2024w11)	Arboviruses (Dengue)	5183/2024	90

Source: Authors' elaboration from state government documents.

Emergency declarations from state governments were obtained in two steps. First, manual retrieval was conducted for all states through systematic searches of their official publications (Diário Oficial). Second, automated searches using the Google API and Programmable Search Engines were applied to all states, restricted to government domain websites. As a robustness check, freedom of information requests were submitted to all state governments. For Amapá, we considered Decree N. 1837/2024 issued in 28/02/2024 which regarded several cities, including the capital Macapá. The earlier Decree N. 1224/2024 issued in 01/02/2024 regarded exclusively the municipality of Oiapoque.

A.1.3.4 State Capitals

The table summarizes which state capitals issued a public health emergency declaration between 2023w40 and 2024w39.

Table 6: **Municipal-specific Emergency Declarations for Dengue (chronological order)**

City (Level)	Type of Decree	Date Issued	Disease	Number	Duration (days)
Florianópolis	Emergency	2023-11-29 (2023w48)	Arboviruses	25785/2023	180
Rio de Janeiro	Emergency	2024-02-02 (2024w5)	Arboviruses (Dengue)	53921/2024	60
Cuiabá	Calamity	2024-02-08 (2024w6)	Arboviruses (Dengue)	10045/2024	60
Belo Horizonte	Emergency	2024-02-16 (2024w7)	Arboviruses	18632/2024	180
Florianópolis	Emergency	2024-02-22 (2024w8)	Arboviruses	26078/2024	180
Natal	Emergency	2024-03-01 (2024w9)	Arboviruses (Dengue)	13024/2024	90
Goiânia	Emergency	2024-03-13 (2024w11)	Arboviruses	786/2024	180
São Paulo	Emergency	2024-03-18 (2024w12)	Arboviruses (Dengue)	63266/2024	-
Porto Alegre	Emergency	2024-04-22 (2024w17)	Arboviruses (Dengue)	22628/2024	-

Source: Authors' elaboration from municipal government documents.

Emergency declarations from municipal governments were obtained in two steps. First, manual retrieval was conducted for all capitals and for the city of Campinas through systematic searches of their official publications (Diário Oficial). Second, automated searches using the Google API and Programmable Search Engines were applied to all capitals, restricted to government domain websites. As a robustness check, freedom of information requests were submitted to all municipal governments.

A.1.4 Primary Health Coverage

Data were obtained from the Primary Health Care (Atenção Primária à Saúde, APS) reporting system, consistent with the National Health Plan (Plano Nacional de Saúde, PNS) 2020–2023. This system monitors population access to health services through the registration and activity of Family Health Strategy (FHS) teams at the household level, administered by the Ministry of Health. Coverage was initially calculated at the municipality level as the ratio between the total number of registered individuals and the total population. In cases where the number of registered individuals exceeded the resident population, coverage values were capped at 100%.

For the regression analyses, this continuous measure was binarized, assigning a value of 1 to localities with coverage greater than or equal to the median and 0 otherwise. Primary Care Coverage is a dummy variable that indicates whether the locality has enrolled its local population at or above the median, and zero otherwise. For states, median Primary Health Coverage was 84·99% in 2023. The percentage of the population enrolled in the Family Health Strategy Program was at or above this level in 13 states. For capitals, the median FHS enrollment was 80·17%. Among capitals, 13 of 27 had median or higher FHS coverage. Localities with higher shares of primary care enrollment would presumably be more concerned that the public health system would become saturated by the influx of cases, thereby increasing the likelihood of a PHED. However, controlling for all other factors, localities with ESF coverage at or above the median were not likely to declare PHEDs using the 95% confidence interval criterion. ESF coverage at the capital and state levels may be interdependent and influence the likelihood of a PHED. For example, ESF was lowest in the capital of Belem (25·33%) of all capitals. In the state of Para, where Belem is the capital, ESF coverage is significantly higher (68·55%), but well below the median for states. Nevertheless, Belem did not declare a PHED, and this may be in part understood considering higher ESF enrollment.

A.1.5 Local Government Political Alignment and Election Cycles

A.1.5.1 Governors Aligned with Lula III Administration

Governors were considered aligned with the Federal Government based on public political positioning at the beginning of the Lula administration. Data were coded using information reported by Poder360.[1] Among the 27 Federation Units (UF), 11 governors were aligned with the federal government, 14 were in opposition, and 2 were non-aligned. Aligned governors included those from PT (Bahia, Ceará, Piauí, Rio Grande do Norte), PSB (Espírito Santo, Maranhão, Paraíba), MDB (Alagoas, Pará), PSD (Sergipe), and SD (Amapá). Opposition or non-aligned governors included those from União Brasil (Amazonas, Goiás, Mato Grosso, Rondônia), PP (Acre, Roraima), PL (Rio de Janeiro, Santa Catarina), PSDB (Mato Grosso do Sul, Pernambuco, Rio Grande do Sul), Novo (Minas Gerais), PSD (Paraná), Republicanos (São Paulo, Tocantins), and MDB in the Federal District.

Table 7: Governor–President Alignment (Brazil, 2023)

UF	Governor	Party	Political Alignment
AC	Gladson Cameli	PP	0
AL	Paulo Dantas	MDB	1
AM	Wilson Lima	União Brasil	0
AP	Clécio Luís	SD	1
BA	Jerônimo Rodrigues	PT	1
CE	Elmano de Freitas	PT	1
DF	Ibaneis Rocha	MDB	0
ES	Renato Casagrande	PSB	1
GO	Ronaldo Caiado	União Brasil	0
MA	Carlos Brandão	PSB	1
MG	Romeu Zema	Novo	0
MS	Eduardo Riedel	PSDB	0
MT	Mauro Mendes	União Brasil	0
PA	Helder Barbalho	MDB	1
PB	João Azevêdo	PSB	1
PE	Raquel Lyra	PSDB	0
PI	Rafael Fonteles	PT	1
PR	Ratinho Jr.	PSD	0
RJ	Cláudio Castro	PL	0
RN	Fátima Bezerra	PT	1
RO	Marcos Rocha	União Brasil	0
RR	Antonio Denarium	PP	0
RS	Eduardo Leite	PSDB	0
SC	Jorginho Mello	PL	0
SE	Fábio Mitidieri	PSD	1
SP	Tarcísio de Freitas	Republicanos	0
TO	Wanderlei Barbosa	Republicanos	0

Source: Authors's elaboration from Poder360

Among the 27 Federation Units (UF), eleven governors were aligned with the federal government, fourteen were in opposition, and two were non-aligned. Aligned governors included those from the Worker's Party, PT. (Bahia, Ceara,

Piauí, Rio Grande do Norte), PSB (Espírito Santo, Maranhão, Paraíba), MDB (Alagoas, Pará), PSD (Sergipe), and SD (Amapá). Opposition or non-aligned governors include those from União Brasil (Amazonas, Goiás, Mato Grosso, Rondônia), PP (Acre, Roraima), PL (Rio de Janeiro, Santa Catarina), PSDB (Mato Grosso do Sul, Pernambuco, Rio Grande do Sul), Novo (Minas Gerais), PSD (Parana), Republicanos (São Paulo, Tocantins), and MDB in the Federal District. After controlling for the number of consecutive weeks of epidemic severity, the alignment between the president and the governor did not influence a state's PHED likelihood using a 95

A.1.5.2 Re-election of Mayors in State Capitals

For capitals, incumbent mayors who were seeking re-election in the 2024 October municipal election were identified with a dummy variable using data from the Supreme Election Court (TSE). 20 incumbents sought re-election. These included the mayors of Belém (PA), Belo Horizonte (MG), Boa Vista (RR), Campo Grande (MS), Florianópolis (SC), Fortaleza (CE), Goiânia (GO), João Pessoa (PB), Macapá (AP), Maceió (AL), Manaus (AM), Porto Alegre (RS), Recife (PE), Rio Branco (AC), Rio de Janeiro (RJ), Salvador (BA), São Luís (MA), São Paulo (SP), Teresina (PI), and Vitória (ES). Conversely, six incumbents were ineligible to run as they were already completing their second consecutive term; these were the mayors of Aracaju (SE), Cuiabá (MT), Curitiba (PR), Natal (RN), Palmas (TO), and Porto Velho (RO). Brasília (DF) is a particular case, in which the DF governor holds the position as mayor; since he is elected in the governor's election, mayoral elections do not apply, and it was coded as not seeking re-election in 2024.

Twenty incumbent mayors were seeking re-election in the forthcoming 2024 October municipal election. These included the mayors of Belem (PA), Belo Horizonte (MG), Boa Vista (RR), Campo Grande (MS), Florianópolis (SC), Fortaleza (CE), Goiânia (GO), Joao Pessoa (PB), Macapá (AP), Maceió (AL), Manaus (AM), Porto Alegre (RS), Recife (PE), Rio Branco (AC), Rio de Janeiro (RJ), Salvador (BA), São Luís (MA), São Paulo (SP), Teresina (PI), and Vitoria (ES). Conversely, six incumbents were ineligible to run as they were already completing their second consecutive term; these were the mayors of Aracaju (SE), Cuiaba (MT), Curitiba (PR), Natal (RN), Palmas (TO), and Porto Velho (RO). Perhaps due to the high number of mayors seeking re-election in 2024, the dummy variable for seeking re-election did not influence a capital's PHED likelihood using a 95% confidence interval criterion.

Table 8: Incumbent Mayors Seeking Reelection (Brazil Capitals, 2024 Election)

Capital	UF	Mayor	Party	Seeking Re-election (2024)
Aracaju	SE	Edvaldo Nogueira	PDT	0
Belém	PA	Edmilson Rodrigues	PSOL	1
Belo Horizonte	MG	Fuad Noman	PSD	1
Boa Vista	RR	Arthur Henrique	MDB	1
Campo Grande	MS	Adriane Lopes	PP	1
Cuiabá	MT	Emanuel Pinheiro	MDB	0
Curitiba	PR	Rafael Greca	PSD	0
Florianópolis	SC	Topazio Neto	PSD	1
Fortaleza	CE	Jose Sarto	PDT	1
Goiânia	GO	Rogerio Cruz	Republicanos	1
João Pessoa	PB	Cicero Lucena	PP	1
Macapá	AP	Dr. Furlan	MDB	1
Maceió	AL	JHC	PL	1
Manaus	AM	David Almeida	Avante	1
Natal	RN	Alvaro Dias	Republicanos	0
Palmas	TO	Cinthia Ribeiro	PSDB	0
Porto Alegre	RS	Sebastiao Melo	MDB	1
Porto Velho	RO	Hildon Chaves	PSDB	0
Recife	PE	Joao Campos	PSB	1
Rio Branco	AC	Tiao Bocalom	PP	1
Rio de Janeiro	RJ	Eduardo Paes	PSD	1
Salvador	BA	Bruno Reis	União Brasil	1
São Luís	MA	Eduardo Braide	PSD	1
São Paulo	SP	Ricardo Nunes	MDB	1
Teresina	PI	Dr. Pessoa	Republicanos	1
Vitória	ES	Lorenzo Pazolini	Republicanos	1
Brasília	DF	Ibaneis Rocha	MDB	0

Source: Authors's elaboration from Poder360

A.2 Analytical Approach

A.2.1 Protocol for Risk Assessment of Dengue Epidemic Potential

The analysis relies on publicly available data on dengue cases in Brazil. The primary dataset consists of weekly epidemiological reports of likely cases and deaths for dengue from 2014 to 2024, aggregated at the municipal level. An auxiliary dataset was used to map calendar weeks to standardized epidemiological weeks. Consistent with arbovirus surveillance practices, an arbovirus epidemiological year was defined as starting on the 40th calendar week of the preceding year and ending on the 39th week of the current year. The analysis uses data from the 40th week of 2014 onwards to avoid including an incomplete arbovirus epidemiological year.

The risk analysis involved building the endemic channel based on Nota Técnica Nº 1/2023-CGARB/DEIDT/SVS/MS and assigning risk levels based on an adaptation of the criteria outlined in the 2025 Plano de Contingência Nacional para Dengue, Chikungunya e Zika.

The cleaned municipal-level data was aggregated to three distinct geographical levels: selected municipalities (state capitals), states, and the national level. For each level, weekly incidence and death rate were calculated.

Incidence is the number of likely cases per 100,000 population. For a given week w and epidemiological year y, the incidence I_w was calculated as:

$$I_w = \frac{C_w}{P_y} \times 100,000$$

where C_w is the number of likely cases in week w, and P_y is the total population for the corresponding geographic unit in year y.

Death rate is the number of deaths per 100,000 population, calculated similarly:

$$D_w = \frac{M_w}{P_y} \times 100,000$$

where M_w is the number of deaths in week w.

A.2.1.1 Endemic Channel

The endemic channel serves as a historical baseline to evaluate the epidemiological situation of the current year. It describes the expected range of disease incidence for each epidemiological week based on past observations. Following the recommendations of Nota Técnica Nº 1/2023-CGARB/DEIDT/SVS/MS, the channel was constructed as follows:

For each epidemiological week w, an endemic channel is constructed using all the available historical years (Y_h). The weekly historical median incidence (M_w) and its 25th percentile ($P_{25,w}$) and 75th percentile ($P_{75,w}$) are calculated from the distribution of incidence values for that week across all years in Y_h. The median for week w, denoted as M_w, is calculated as:

$$M_w = \begin{cases} i_{((n+1)/2)} & \text{if } n \text{ is odd} \\ \frac{1}{2}(i_{(n/2)} + i_{(n/2+1)}) & \text{if } n \text{ is even} \end{cases}$$

The p-th percentile for week w, $P_{p,w}$, is calculated using a linear interpolation method based on its rank, $k = \frac{p}{100}(n-1)+1$. Let $i = \lfloor k \rfloor$ be the integer part of the rank and $f = k - i$ be the fractional part. The value is:

$$P_{p,w} = i_{(i)} + f \cdot (i_{(i+1)} - i_{(i)})$$

The interval between the lower ($P_{25,w}$) and upper ($P_{75,w}$) bounds defines the endemic channel, representing the expected range of incidence for week w based on historical data.

A.2.1.2 Risk Level Classification

Then, the risk analysis function assigns a risk level for each week of the analysis year by comparing its current incidence and mortality data against the endemic channel and a set of rules adapted from the 2025 Plano de Contingência Nacional para Dengue, Chikungunya e Zika. The adaptation was necessary due to the unavailability of certain real-time clinical and virological data.

For Mobilization, the official criteria include: incidence above the historical median for four consecutive weeks; nowcasting data indicating an increase; RT-PCR positivity greater than 20% for four weeks; and detection of serotype inversion.

In the implementation, mobilization is defined as incidence between the historical median and the upper bound for four consecutive weeks. The rationale for this adaptation is that nowcasting, RT-PCR, and serotype data were not available in the public dataset, so the core epidemiological indicator was retained.

For Alert, the official criteria include: incidence above the upper bound for four consecutive weeks; nowcasting above the upper bound for four consecutive weeks; severe cases greater than 1% of probable cases; increased deaths for four consecutive weeks; lethality greater than 0.05%; and new serotype proportion greater than or equal to 20%.

The implemented criteria consider any of the following: incidence above the upper bound for four consecutive weeks; increased deaths for four consecutive weeks; or lethality greater than 0.05%. This adaptation was necessary because data on nowcasting, clinical severity, and serotype circulation were unavailable. Key incidence, mortality, and lethality triggers were retained.

For Epidemic, the official criteria include: exponential increase in incidence above the upper bound; exponential increase in nowcasting; severe cases greater than or equal to 1% of probable cases; and increased deaths for more than four consecutive weeks. Nowcasting and severe case data were unavailable.

In the implemented criteria, epidemic is defined by either a logarithmic increase in incidence while above the upper bound or increased deaths for five consecutive weeks. In the implementation,“exponential increase” was operationalized as week-over-week increase in the natural logarithm of incidence, or increased deaths for five consecutive weeks. so “exponential increase” was operationalized using a log-linear comparison, and “more than four weeks” was implemented as five weeks.

The implemented risk levels are:

- **Learning**: The first five weeks of the analysis.
- **Normality**: The baseline status.

- **Mobilization**: Triggered when incidence exceeds the historical median (M_w) but remains below the upper bound ($P_{75,w}$) for four consecutive weeks.
- **Alert**: Triggered if any of the following conditions are met:
  - The weekly case fatality rate (deaths / cases) exceeds 0.05%.
  - Incidence exceeds the upper bound of the endemic channel ($P_{75,w}$) for four consecutive weeks.
  - Week-over-week increases in death rate for four consecutive weeks.
- **Epidemic**: Triggered if any of the following conditions are met:
  - An exponential increase in incidence (operationalized as a week-over-week increase in the natural logarithm of incidence) occurs while incidence is already above the upper bound ($P_{75,w}$).
  - Week-over-week increases in death rate for more than four consecutive weeks (implemented as five weeks).

A.2.2 Summary Table of Risk Assessment in Epidemiological Year 2024 (2023w40 - 2024w39)

'Max. Weeks' refers to the maximum consecutive number of weeks under a given risk level.

A.2.2.1 Country Level

Table 9: List of First Week and Max. consecutive weeks for level 2 and 3 - Country Level

Loc.	First Week Alert (level 2)	Max. Weeks Alert (level 2)	First Week Epidemic (level 3)	Max. Weeks Epidemic (level 3)	Max. Weeks (level 2 or 3)
Brazil	2023w44	19	2023w45	11	48

Source: Authors' elaboration based on data from the Notifiable Diseases Information System (SINAN)

A.2.2.2 State Level

Table 10: List of First Week and Max. consecutive weeks for level 2 and 3 - State Level

Loc.	First Week Alert (level 2)	Max. Weeks Alert (level 2)	First Week Epidemic (level 3)	Max. Weeks Epidemic (level 3)	Max. Weeks (level 2 or 3)
AC	2024w1	1	2023w50	3	5
AL	2024w10	3	2024w2	4	10
AM	2024w1	2	2023w46	3	15
AP	2024w3	5	2023w45	7	40
BA	2023w44	16	2023w45	12	30
CE	2024w13	1	No Epidemic	0	1
DF	2024w1	18	2023w44	9	35
ES	2023w44	9	2023w45	10	39
GO	2023w47	13	2023w45	5	36
MA	2023w49	5	2023w46	7	14
MG	2023w44	21	2023w45	20	48
MS	2023w47	8	No Epidemic	0	8
MT	2024w3	5	2023w44	4	24
PA	2024w9	8	2024w2	7	38
PB	2024w4	1	2023w50	4	6
PE	2023w46	1	2024w8	5	10
PI	2024w3	3	2023w46	8	26
PR	2023w44	10	2023w45	20	48
RJ	2023w44	14	2023w45	14	48
RN	2023w45	2	2024w2	4	8

RO	2023w44	4	2023w45	1	5
RR	2024w24	6	2024w5	2	8
RS	2023w44	9	2023w45	15	48
SC	2023w44	11	2023w45	14	48
SE	2024w6	2	2023w44	2	2
SP	2023w44	18	2023w45	21	48
TO	2023w48	2	No Epidemic	0	2

Source: Authors' elaboration based on data from the Notifiable Diseases Information System (SINAN)

A.2.2.3 State Capitals Level

Table 11: List of First Week and Max. consecutive weeks for level 2 and 3 - State Capital Level

Loc.	First Week Alert (level 2)	Max. Weeks Alert (level 2)	First Week Epidemic (level 3)	Max. Weeks Epidemic (level 3)	Max. Weeks (level 2 or 3)
ARACAJU (SE)	2024w6	1	2023w44	3	3
BELEM (PA)	2024w9	8	2024w1	8	39
BELO HORIZONTE (MG)	2023w45	10	2023w44	16	48
BOA VISTA (RR)	2024w21	4	2024w5	3	11
BRASILIA (DF)	2024w1	18	2023w44	9	35
CAMPO GRANDE (MS)	2024w4	2	No Epidemic	0	2
CUIABA (MT)	2024w6	3	2024w1	2	5
CURITIBA (PR)	2024w3	10	2023w48	6	41
FLORIANOPOLIS (SC)	2023w44	13	2023w45	6	48
FORTALEZA (CE)	2024w22	1	No Epidemic	0	1
GOIANIA (GO)	2023w48	5	2024w8	2	34
JOAO PESSOA (PB)	2024w11	3	2023w47	3	10
MACAPA (AP)	2024w7	2	2024w2	5	38
MACEIO (AL)	2023w49	3	2023w46	6	28
MANAUS (AM)	2023w48	2	2023w50	6	10
NATAL (RN)	2024w13	2	2023w45	6	8
PALMAS (TO)	2023w48	1	No Epidemic	0	1
PORTO ALEGRE (RS)	2023w44	7	2023w45	14	48
PORTO VELHO (RO)	2023w45	1	2023w44	2	3
RECIFE (PE)	2024w12	2	2024w6	6	12
RIO BRANCO (AC)	2023w44	2	2023w45	3	15
RIO DE JANEIRO (RJ)	2023w44	10	2023w45	7	32
SALVADOR (BA)	2023w45	4	2023w44	5	17
SAO LUIS (MA)	2024w9	2	2023w46	2	10

SAO PAULO (SP)	2023w44	18	2023w45	20	48
TERESINA (PI)	2024w10	6	2023w46	2	12
VITORIA (ES)	2023w44	5	2023w45	4	37

Source: Authors' elaboration based on data from the Notifiable Diseases Information System (SINAN)

A.2.3 Visualizing Risk Assessment and Emergency Declarations in 2024

A.2.3.1 Country-wide Risk Assessment of Dengue Outbreak and Emergency Declarations in 2023w40-2024w39

Figure 13: Country-wide Risk Assessment of Dengue Outbreak and Emergency Declarations, 2023w40 to 2024w39

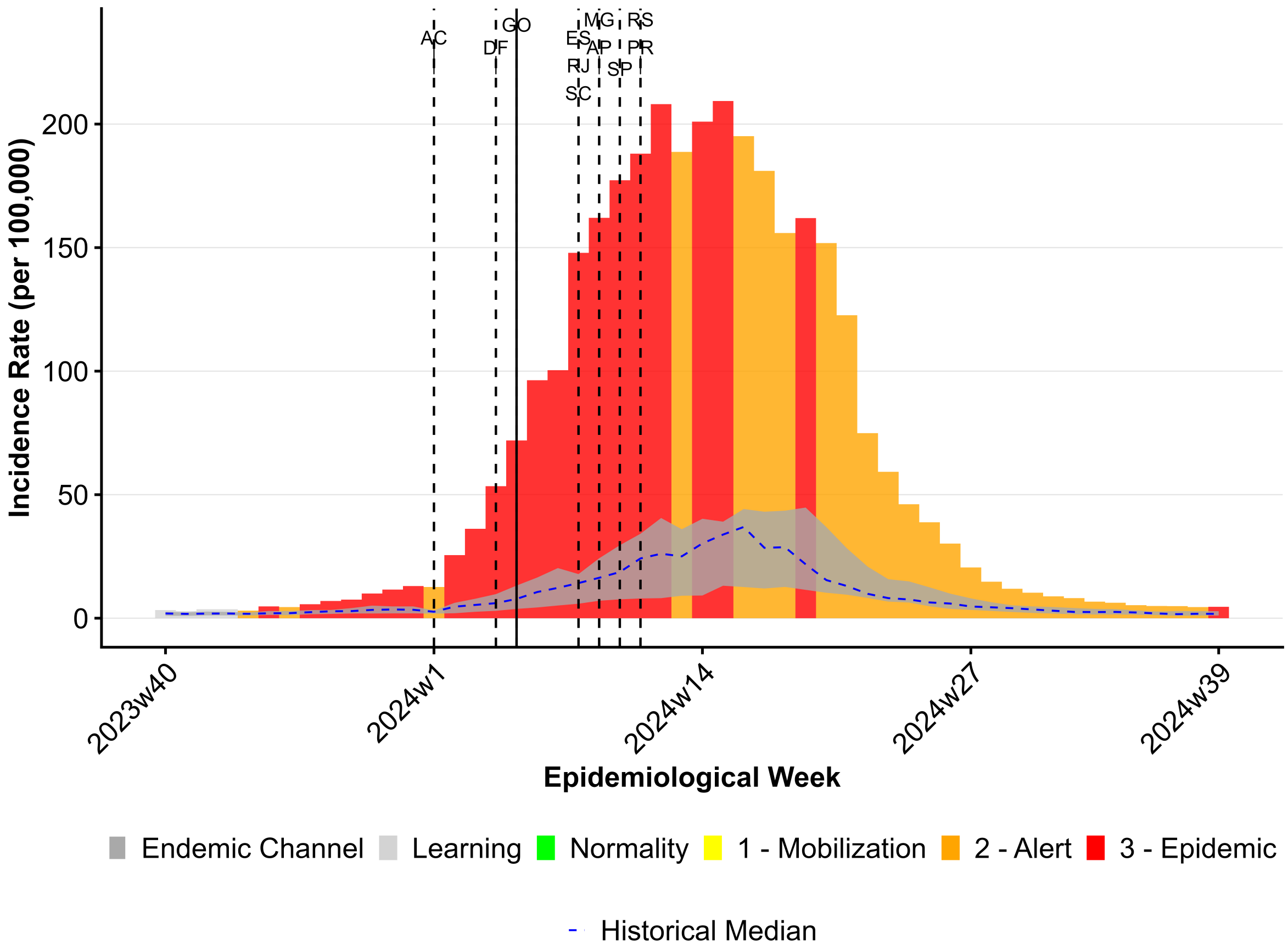

Source: Authors' elaboration based on data from the Notifiable Diseases Information System (SINAN).
Note: Solid line indicates the creation of the COE at the Federal Level. Dashed lines indicate Emergency declarations by States.

A.2.3.2 State-level Risk Assessment of Dengue Outbreak and States Emergency Declarations, 2023w40-2024w39

Figure 14: Risk Assessment of Dengue Outbreak and States Emergency Declarations, 2023w40 to 2024w39

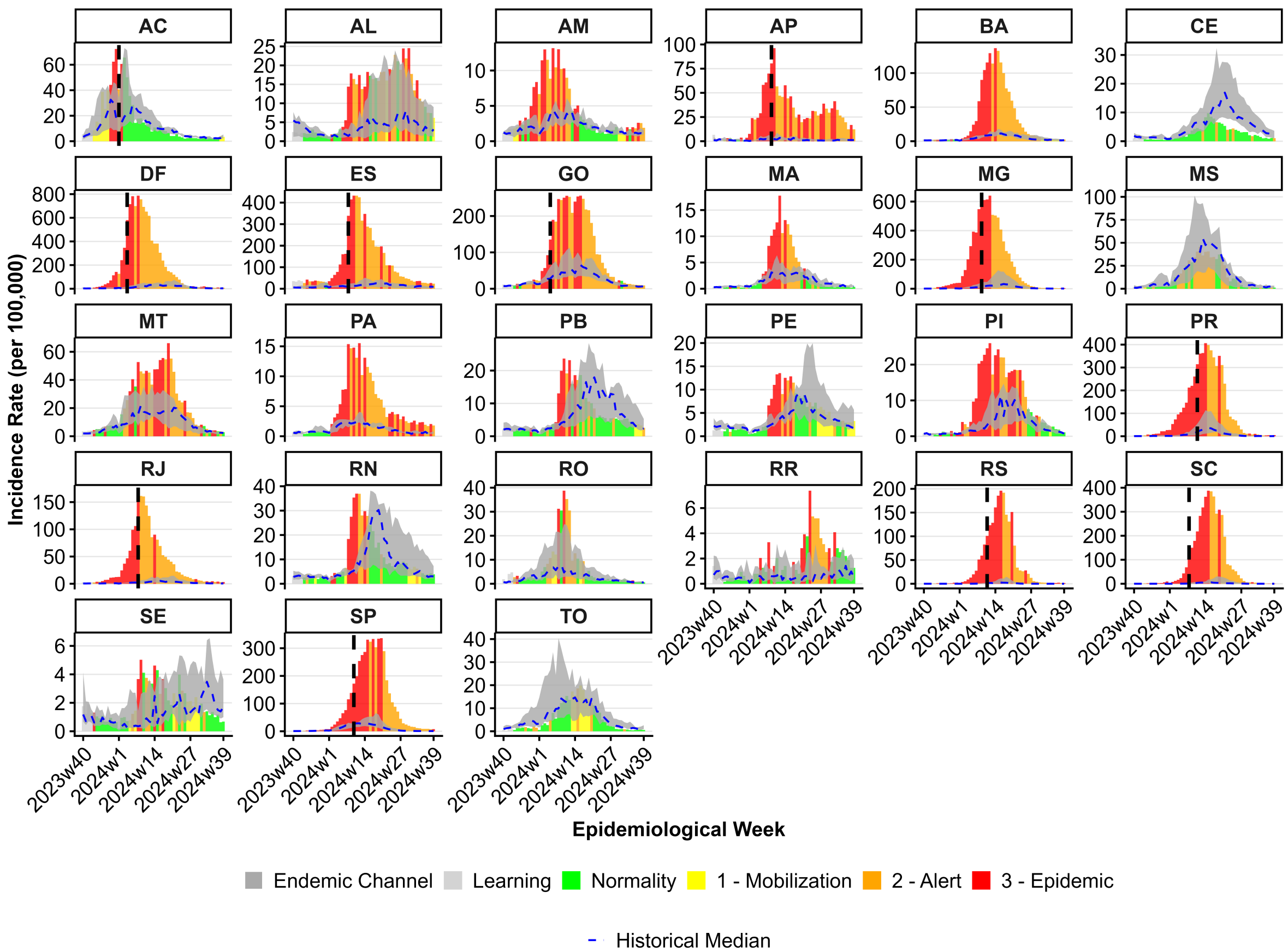

Source: Authors' elaboration based on data from the Notifiable Diseases Information System (SINAN).
Note: Dashed lines indicate Emergency declarations by States.

A.2.3.3 State Capital-level Risk Assessment of Dengue Outbreak and Emergency Declarations, 2023w40-2024w39

Figure 15: Municipality-level Risk Assessment of Dengue Outbreak and Emergency Declarations, 2023w40 to 2024w39

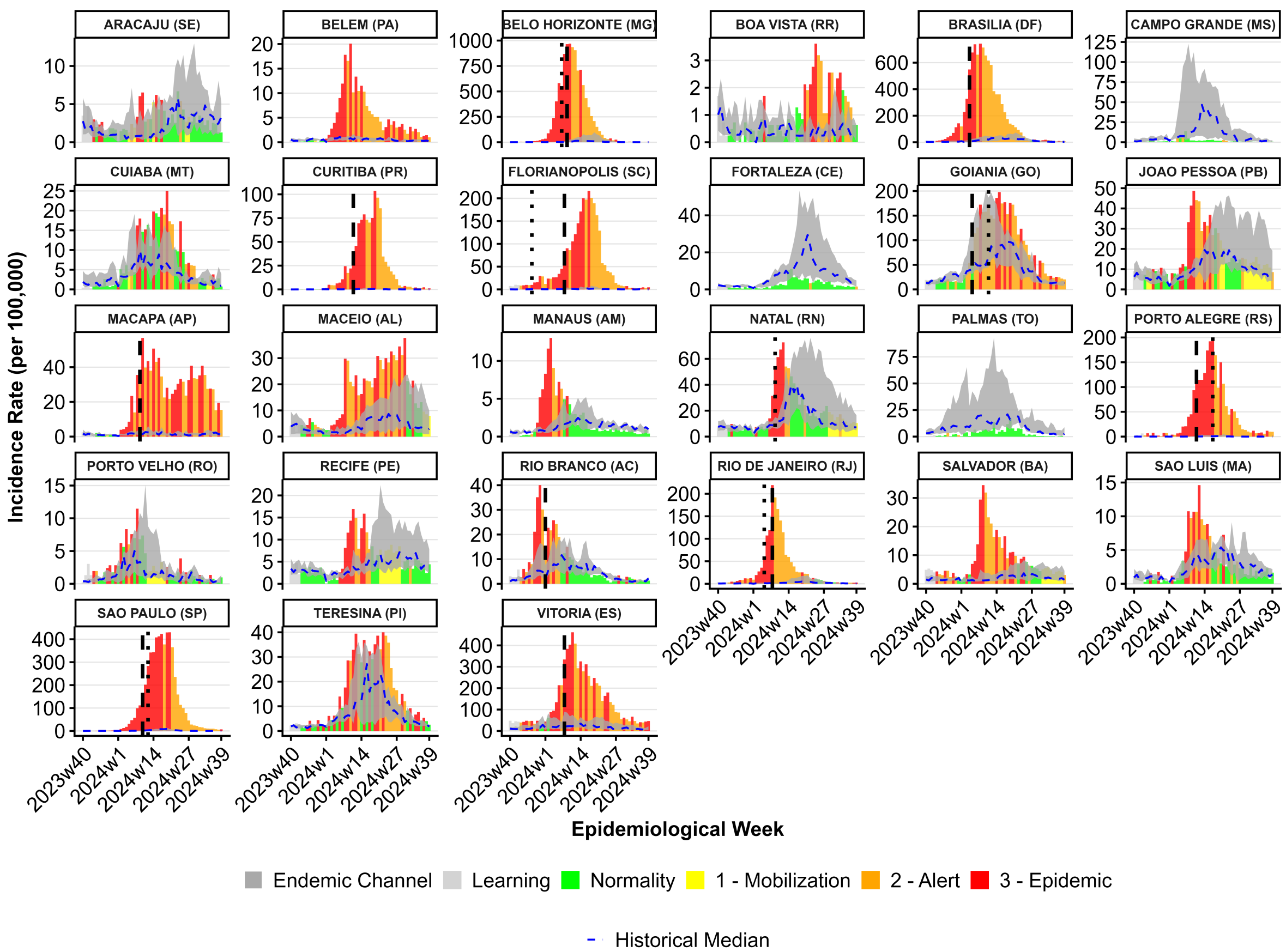

Source: Authors' elaboration based on data from the Notifiable Diseases Information System (SINAN).
Note: Dashed lines indicate Emergency declarations by States. Dotted lines indicate Emergency declarations by Municipalities.

A.2.3.4 Map of First Epidemic Week, 2023w40-2024w39, States and Capitals

Figure 16: Map of First Epidemic Week, 2023w40-2024w39, States and Capitals

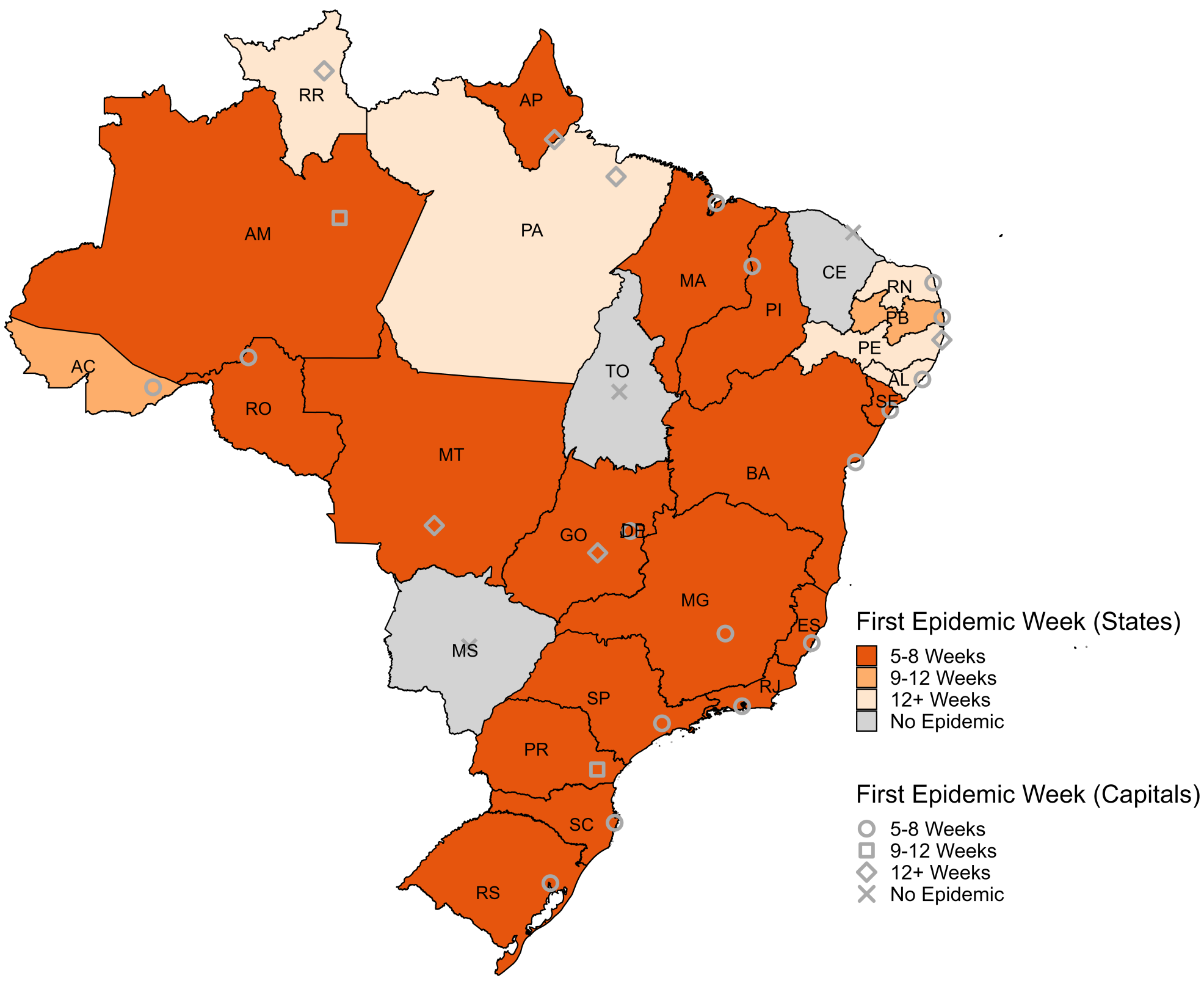

Source: Authors' elaboration based on data from the Notifiable Diseases Information System (SINAN)

A.2.3.5 Map of Lag between First Epidemic Week and Emergency declaration, 2023w40-2024w39, States and Capitals

Figure 17: Map of Lag between First Epidemic Week and Emergency declaration, 2023w40-2024w39, States and Capitals

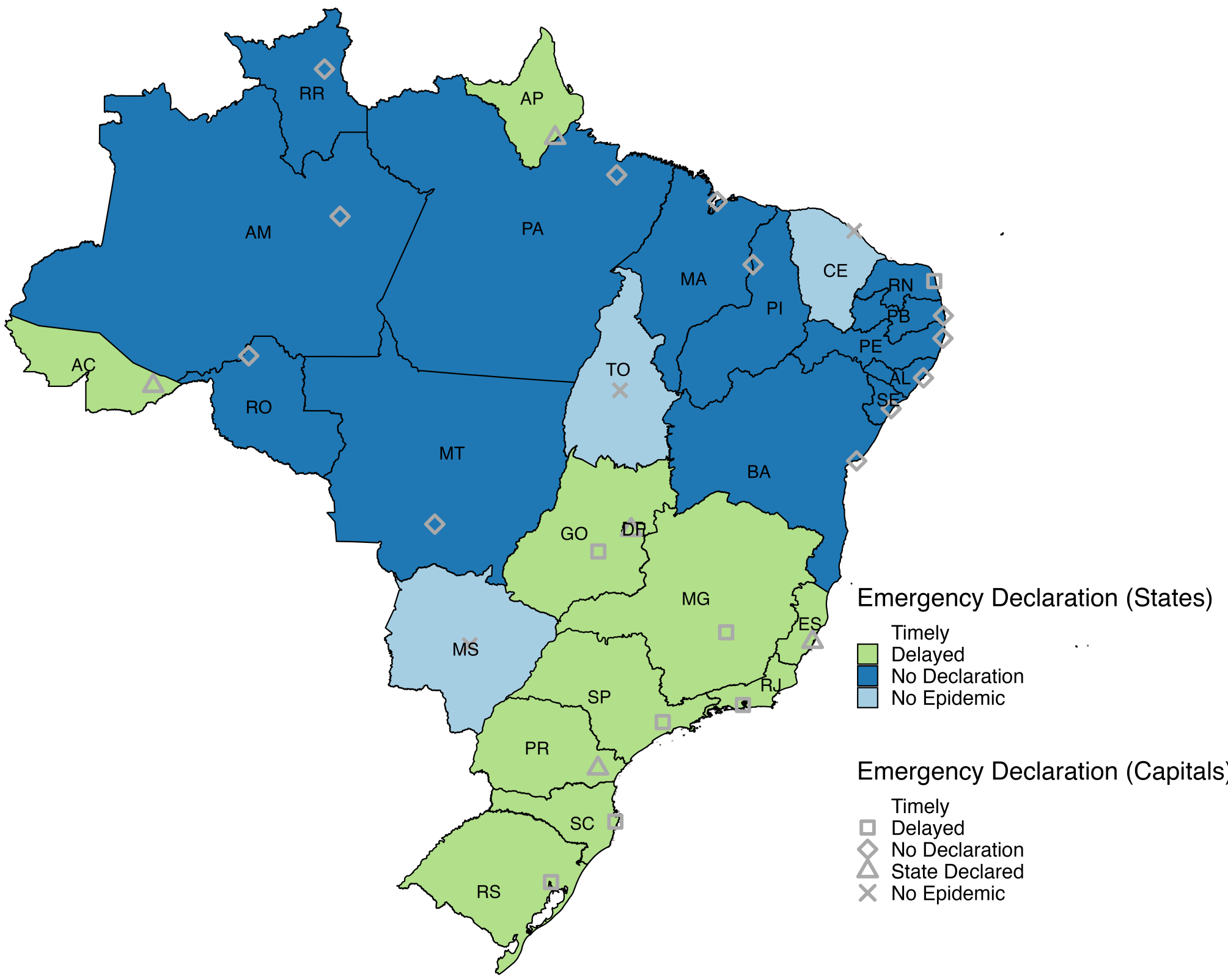

Source: Authors' elaboration based on data from the Notifiable Diseases Information System (SINAN)

A.2.3.6 Correlation Between Max. Consecutive Weeks under 'Epidemic' and 'Alert' Risk Level and Emergency Declaration, 2023w40-2024w39, States

Figure 18: Correlation Between Max. Consecutive Weeks under 'Epidemic' and 'Alert' Risk Level and Emergency Declaration, 2023w40-2024w39, States

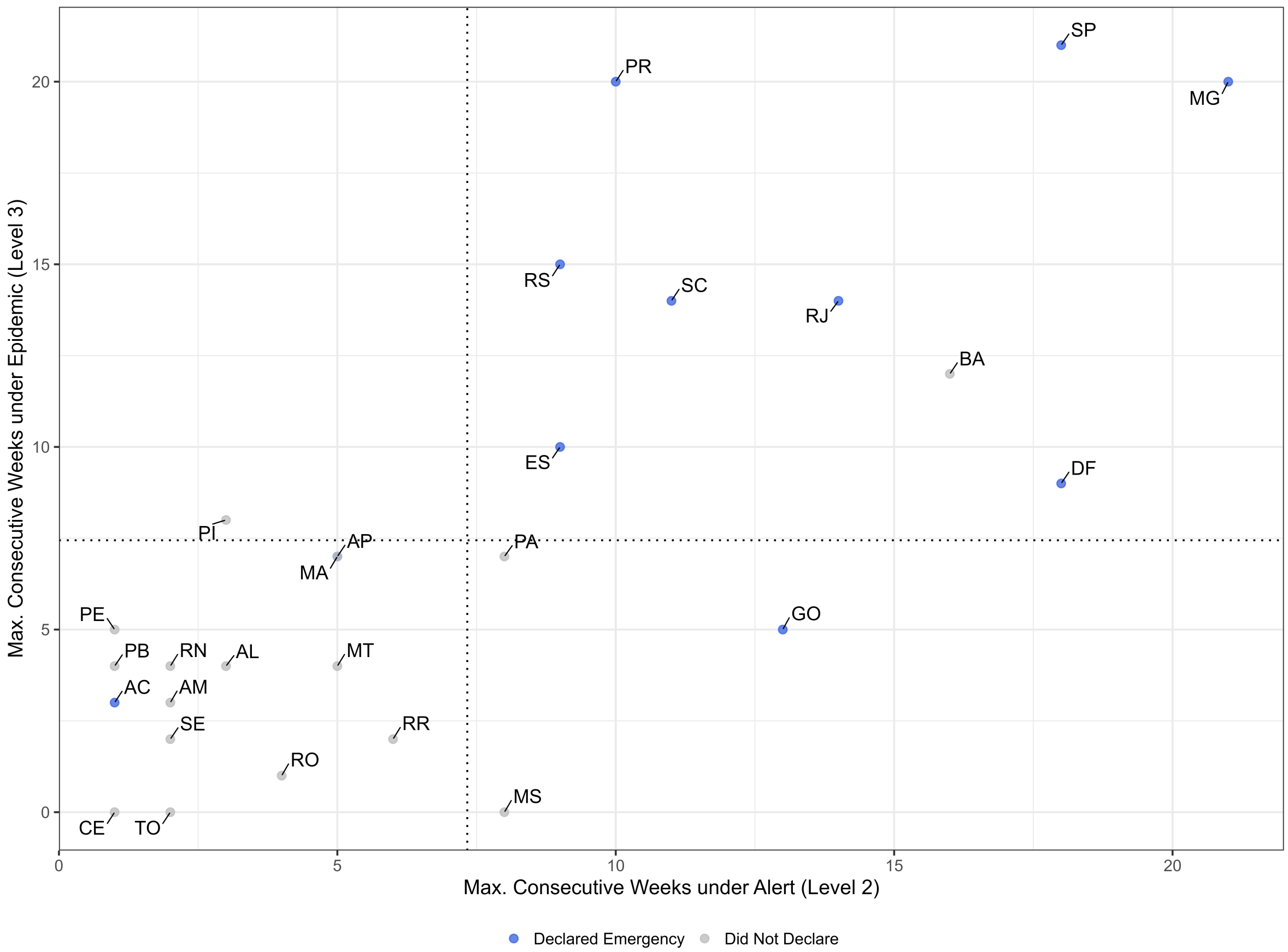

Source: Authors' elaboration based on data from the Notifiable Diseases Information System (SINAN).
Note: Dotted lines indicate average values.

A.2.3.7 Correlation Between Max. Consecutive Weeks under 'Epidemic' and 'Alert' Risk Level and Emergency Declaration, 2023w40-2024w39, Capitals

Figure 19: Correlation Between Max. Consecutive Weeks under 'Epidemic' and 'Alert' Risk Level and Emergency Declaration, 2023w40-2024w39, Capitals

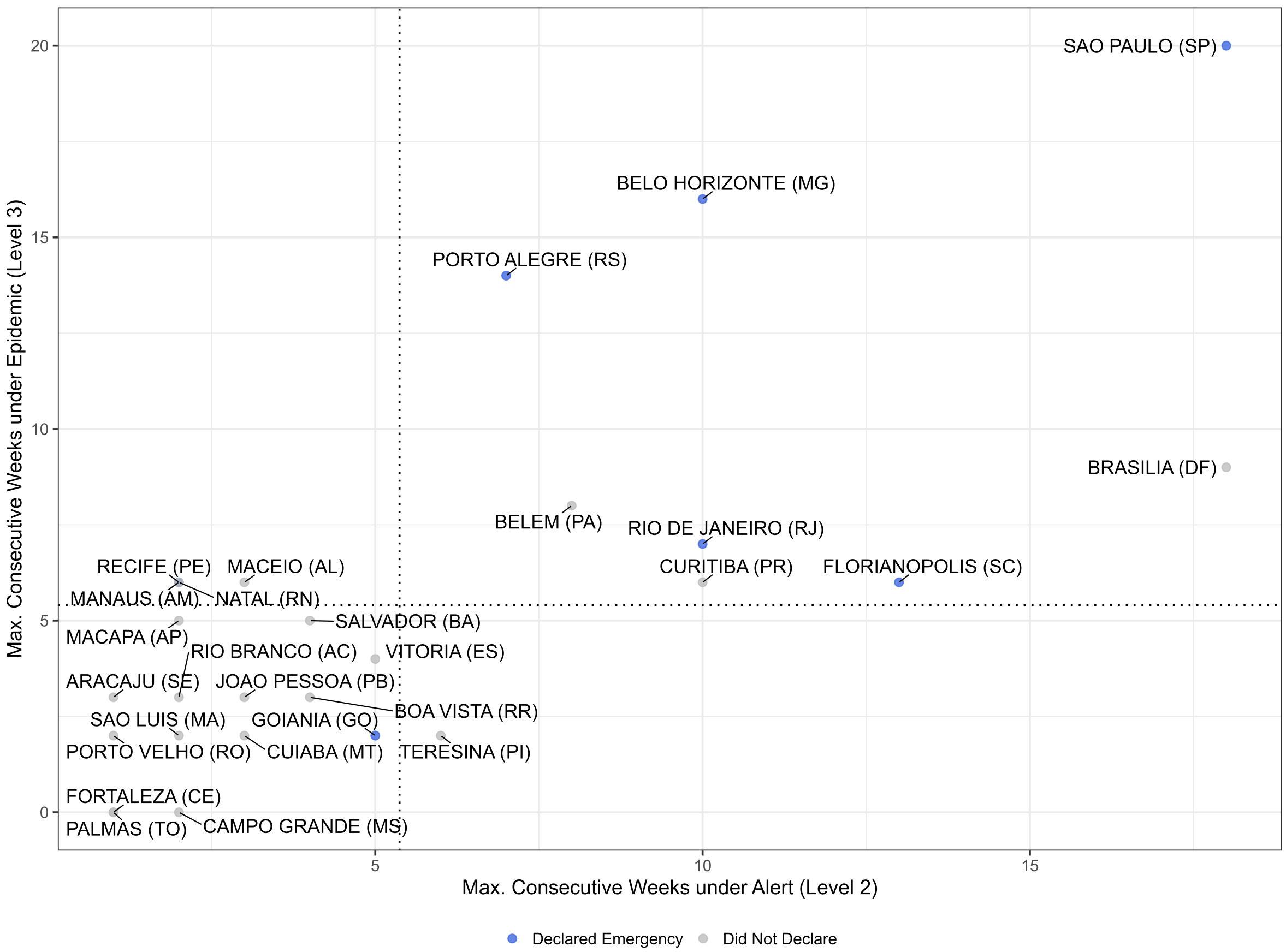

Source: Authors' elaboration based on data from the Notifiable Diseases Information System (SINAN).
Note: Dotted lines indicate average values.

A.2.4 Classification of Emergency Declaration Timeliness of State and States Capitals for 2024 Epidemic

Table 12: Final Classification of State Capitals and States for 2024 Epidemic

Cases	Capitals	States
Preemptive<br>(Epiweek Level 3_t > Epiweek Emergency Declaration$_t$)	None	None
Timely<br>(Epiweek Level 3_t = Epiweek Emergency Declaration$_t$)	None	None
Delayed<br>(Epiweek Level 3_t < Epiweek Emergency Declaration$_t$)	Belo Horizonte (MG)<br>Brasília (DF)[a]<br>Curitiba (PR)[a]<br>Florianópolis (SC)<br>Goiânia (GO)<br>Macapá (AP)[a]<br>Natal (RN)<br>Porto Alegre (RS)<br>Rio Branco (AC)[a]<br>Rio de Janeiro (RJ)<br>São Paulo (SP)<br>Vitória (ES)[a]	Acre<br>Amapá<br>Distrito Federal<br>Espírito Santo<br>Goiás<br>Minas Gerais<br>Paraná<br>Rio de Janeiro<br>Rio Grande do Sul<br>Santa Catarina<br>São Paulo
No Declaration<br>(Despite reaching Level 3, no emergency declaration was issued)	Aracaju (SE)<br>Belém (PA)<br>Boa Vista (RR)<br>Cuiabá (MT)<br>João Pessoa (PB)<br>Maceió (AL)<br>Manaus (AM)<br>Porto Velho (RO)<br>Recife (PE)<br>Salvador (BA)<br>São Luís (MA)<br>Teresina (PI)	Alagoas<br>Amazonas<br>Bahia<br>Maranhão<br>Mato Grosso<br>Pará<br>Paraíba<br>Pernambuco<br>Piauí<br>Rio Grande do Norte<br>Rondônia<br>Roraima<br>Sergipe
No Epidemic<br>(Risk levels did not reach Level 3)	Campo Grande (MS)<br>Fortaleza (CE)<br>Palmas (TO)	Ceará<br>Mato Grosso do Sul<br>Tocantins

[a] Capital did not issue a self-declaration; classified as delayed based on state-level (supra) declaration.

A.2.5 Epidemic Severity and Public Health Declarations

A.2.5.1 Empirical Strategy

We conducted a series of logistic regression analyses at two distinct administrative levels (states [N=27] and capital cities [N=27]) to assess the correlation between increased risk levels and the declaration of a public health emergency. We employed Firth's bias-reduced logistic regression in R (v. 4.5.2), as implemented in the **logistf** package (v 1.26.1).

In all six models, the dependent variable in is a binary indicator coded as 1 if the respective administrative unit (state or capital city) issued a dengue-related public health emergency declaration during the 2024 epidemiological year, and 0 if no such declaration was made. Across all models, the independent variables of theoretical interest are the measures of risk duration, including the maximum number of consecutive weeks at 'Level 2 - Alert', the maximum number of consecutive weeks at 'Level 3 - Epidemic', and the maximum number of consecutive weeks at either 'Level 2' or 'Level 3'.

All models include Primary Health Coverage as a control variable. Coverage was initially calculated at the municipality level as the ratio between the total number of registered individuals and the total population. In cases where the number of registered individuals exceeded the resident population, coverage values were capped at 100%. For the regression analyses, this continuous measure was binarized, assigning a value of 1 to localities with coverage greater than or equal to the median and 0 otherwise.

For the state-level analyses (Models 1-3), the control variables also include President-Governor Alignment. President-Governor Alignment is a binary variable coded 1 for states where the governor was politically aligned with the president and 0 for those in opposition or with no clear position.

For the municipal-level analyses (Models 4–6) focusing on the 27 state capitals, the control variables include: (1) a variable for Mayor Seeking Reelection, coded 1 if the incumbent mayor ran for reelection in 2024 and 0 otherwise, based on data from the Supreme Electoral Court; and (2) a variable coded 1 if the state government declared an emergency before the capital city did (or if the state declared and the city never did), and 0 otherwise.

A.2.5.2 Regression Results

Table 13: Firth's Bias-Reduced Logistic Regression: Dependent Variable – Emergency Declaration

	Model 1			Model 2			Model 3		
Characteristic	OR	95% CI	p-value	OR	95% CI	p-value	OR	95% CI	p-value
Panel A: States									
Max Consecutive Weeks Level 2 (Alert)	1.23	1.05, 1.56	0.009						
Max Consecutive Weeks Level 3 (Epidemic)				1.35	1.10, 2.16	<0.001			
Max Consecutive Weeks Level 2 or 3							1.12	1.05, 1.27	<0.001
Primary Care Coverage (Above Median)	0.53	0.08, 3.38	0.50	0.42	0.04, 3.83	0.43	0.52	0.04, 5.37	0.58
President–Governor Alignment	0.35	0.04, 2.30	0.27	0.25	0.01, 1.93	0.19	0.17	0.01, 1.55	0.12

	Model 4			Model 5			Model 6		
Characteristic	OR	95% CI	p-value	OR	95% CI	p-value	OR	95% CI	p-value
Panel B: Capitals									
Max Consecutive Weeks Level 2 (Alert)	1.47	1.11, 2.25	0.002						
Max Consecutive Weeks Level 3 (Epidemic)				1.35	1.06, 2.06	0.010			
Max Consecutive Weeks Level 2 or 3							1.13	1.05, 1.27	<0.001
Primary Care Coverage (Above Median)	0.30	0.03, 1.92	0.21	0.87	0.16, 5.22	0.87	0.75	0.08, 6.92	0.78
Mayor Seeking Reelection	0.59	0.05, 5.71	0.64	0.70	0.11, 4.56	0.71	0.17	0.00, 2.38	0.20

N = 27 for both panels. CI = Confidence Interval. OR = Odds Ratio. Panel A controls for primary care coverage and president–governor alignment. Panel B controls for primary care coverage and whether the mayor was seeking reelection.
Source: Authors' elaboration based on data from the Notifiable Diseases Information System (SINAN).

Notes

1. Poder360. "Lula takes office with support of 11 governors and opposition from 14." Available at: https://www.poder360.com.br/brasil/lula-assume-com-o-apoio-de-11-governadores-e-oposicao-de-14/. Last accessed on November 5th, 2025.
